# Disentangling Seasonality from Co-Occurrence: Anomaly-Driven Networks of Migratory Waterbirds

**DOI:** 10.3390/biology15070522

**Published:** 2026-03-25

**Authors:** Chien-Hen Hung, Pei-Fen Lee

**Affiliations:** 1 Institute of Ecology and Evolutionary Biology, National Taiwan University, Taipei 10617, Taiwan; 2 Taiwan Power Research Institute, Taiwan Power Company, Taipei 10046, Taiwan

**Keywords:** coastal wetland, migratory waterbirds, time series, phenology, anomaly-based associations, abundance anomaly, species associations, ecological network, Spearman correlation, false discovery rate (FDR)

## Abstract

Understanding how migratory waterbird species co-vary through time can reveal guild structure and guide monitoring in dynamic coastal wetlands, yet seasonal phenology can inflate simple co-occurrence signals. Here, we used standardized monthly bird counts from Yongan Wetland, Taiwan (36 survey months across two survey blocks: November 2014 and January–August 2015, and October 2016–December 2018) to infer de-seasonalized interspecific associations. We analyzed 50 regularly recorded species and a focal subset of 13 shorebirds and ducks. For each species, we transformed raw counts to monthly anomalies that remove recurrent seasonal patterns, then quantified pairwise Spearman correlations and controlled multiple testing using Benjamini–Hochberg FDR (q ≤ 0.05) to construct association networks. The anomaly-based network revealed strong guild structure: positive links were concentrated within dabbling ducks and within shorebirds, consistent with shared habitat use and foraging regimes, whereas negative links were fewer and suggested potential niche partitioning or spatiotemporal segregation. Robustness analyses (moving-block bootstrap stability, a circular-shift null comparison, and log-transformed anomaly sensitivity) supported that the main network patterns were unlikely to arise from chance alignment. Our framework provides a transparent, time-series–based approach for disentangling phenology from association inference, offering a practical framework for wetland monitoring and hypothesis generation about waterbird community dynamics.

## Simple Summary

Coastal wetlands are vital “service stations” for migratory waterbirds, but these communities change strongly with the seasons. Because many species peak at similar times during migration and wintering, simple co-occurrence patterns can be misleading: species may appear linked only because they share the same seasonal timing. In this study, we used long-term monthly bird counts from Yongan Wetland (Taiwan) to separate predictable seasonal cycles from unexpected month-to-month changes, and then examined which species tended to rise and fall together beyond the shared seasonal pattern. This approach revealed a clear community signal: ducks and shorebirds often formed distinct groups of synchronized dynamics, while some pairs showed consistent negative relationships that may reflect different habitat use or timing. By summarizing these patterns as an association network, we provide an intuitive way to track community reorganization over time and to identify a “core” set of robust links that persist across robustness checks. The results offer a practical framework for wetland monitoring that complements single-species trends and helps managers interpret community-level change in strongly seasonal migratory systems.

## 1. Introduction

Coastal wetlands are among the most rapidly changing ecosystems worldwide, yet they remain indispensable nodes along migratory flyways where waterbirds refuel, overwinter, and reorganize their habitat use across seasons. Global wetland loss and degradation have already translated into measurable population declines in migratory waterbirds that depend on a chain of functioning stopover and non-breeding sites [1,2]. Recent large-scale syntheses further show that habitat change in shorebird-relevant coastal wetlands remains widespread across flyways, with particularly severe losses and degradation in parts of the East Asian–Australasian Flyway [3,4]. In addition, recent work indicates that direct human-caused mortality can compound habitat-driven decline in migratory shorebirds, emphasizing that contemporary conservation pressure extends beyond habitat loss alone [5]. Because management actions and habitat changes in coastal wetlands can propagate through entire assemblages, conservation monitoring increasingly needs tools that move beyond single-species trends and ask how communities are structured and restructured through time. Ecological network thinking provides one such lens by treating species as components of an interconnected system rather than isolated response variables [6]. In this study, we use long-term monthly count time series to quantify interspecific associations as a community-level signal that can support hypothesis generation and monitoring, while explicitly recognizing that association patterns are proxies rather than direct proof of interaction mechanisms [7,8].

### 1.1. Study Context: Coastal Wetlands and Migratory Waterbirds; Why Interspecific Associations Matter

Coastal wetlands—including tidal flats, lagoons, and human-managed habitats such as saltpans—provide spatially concentrated food resources and shallow-water foraging opportunities that can sustain high densities of migratory waterbirds during stopover and non-breeding periods. However, these habitats are under exceptional pressure from land reclamation, hydrological alteration, and broader environmental change, contributing to pronounced declines in migratory shorebirds along major flyways when critical staging sites are lost or degraded [1,2]. Recent studies have further shown that losses are not limited to total wetland area, but also include declines in habitat suitability, habitat connectivity, and the availability of high-quality stopover and non-breeding habitat within coastal mosaics [4,9]. In such systems, community composition is not static: it is repeatedly rebuilt by migration phenology, seasonal resource pulses, and shifting habitat accessibility. This dynamism means that conservation-relevant change can manifest not only as the rise or fall of individual species, but also as **reorganization in how species co-vary through time**—a signal that routine monitoring can capture if analyzed appropriately.

To interpret such temporal reorganization, we distinguish between two related but different signals. Raw monthly counts describe co-occurrence in time, meaning that species may appear synchronized simply because they share the same seasonal schedule. By contrast, anomaly-based series describe co-fluctuation around each species’ typical seasonal baseline and therefore provide the basis for the ecological associations examined here. In this study, we use **ecological association** to mean a temporal statistical association between species after accounting for regular seasonality, not direct evidence of species interaction. Network approaches formalize this anomaly-based perspective by representing species as nodes and association signals as edges, enabling questions about community organization (e.g., clustering, hub-like species, or bridging roles) that single-species analyses cannot address directly [6]. At the same time, these patterns must be interpreted conservatively: simple temporal co-occurrence in raw counts does not, by itself, demonstrate ecological interaction and can reflect common responses to environment or seasonality [7,8]. In contrast, anomaly-based ecological associations can still provide actionable value for wetland monitoring by highlighting consistent co-fluctuation patterns, identifying candidate guilds that track habitat states, and generating testable hypotheses about community-level responses to habitat change.

### 1.2. Problem/Gap: Seasonality Confounds Co-Occurrence; Need De-Seasonalized Association Inference

Seasonal migration imposes strong, repeatable phenological structure on coastal wetland bird assemblages: many species show predictable **abundance peaks** during arrival, wintering, and spring departure, with abundance trajectories that are largely synchronized to regional climate and migratory timing. As a result, raw monthly counts can overstate ecological association because species with similar seasonal schedules may appear strongly correlated even when their departures from the seasonal baseline are unrelated. For example, two species may both peak in winter and thus show a strong raw-count correlation, yet one may be above its typical winter level in a given year while the other is below it. This caution is particularly important in migratory wetland assemblages, where rapid habitat change and shifting stopover conditions can modify both seasonal timing and apparent co-occurrence structure across years [3,4,9]. More generally, time-series analyses have long warned that correlations between temporally structured series can become “nonsense correlations” if dominant cyclic components are not addressed [10].

This problem creates a practical knowledge gap for migratory bird monitoring and community inference: we still lack an operational and transparent workflow for inferring ecological associations from multi-year monthly abundance data while explicitly controlling for seasonal phenology as the dominant confounder. Many community analyses either (i) collapse data into broad seasonal summaries, which can mask within-season covariance and weaken inference, or (ii) analyze full time series directly, where shared seasonality dominates the signal. A defensible alternative is to first remove the expected seasonal component and then evaluate associations on de-seasonalized anomalies—that is, deviations from the typical phenological baseline—so that inferred links reflect co-fluctuations beyond shared seasonality [11]. In this study, we treat de-seasonalization as the necessary first step toward interpretable association inference in a strongly seasonal migratory system, and we then test whether the resulting network structure is robust across transformation choice and null-model comparison [8].

### 1.3. Approach Overview

Our workflow had four steps. First, we summarized seasonal phenology from the monthly count record to establish the strength of seasonal structure in the assemblage. Second, we removed each species’ recurring month-of-year baseline to construct anomaly series that represent departures from the typical seasonal expectation. Third, we quantified pairwise Spearman correlations for both raw counts and anomalies and corrected for false discovery using the Benjamini–Hochberg false discovery rate (FDR) across an assemblage of 50 species [12,13]. Fourth, we summarized results for a focal group of 13 species and evaluated robustness with block resampling, a circular-shift null comparison, and a comparison between log-transformed and untransformed anomaly series. This workflow was designed to distinguish shared seasonal timing from residual co-fluctuation, not to infer direct interaction [14].

### 1.4. Objectives and Hypotheses

Building on this motivation, we set two complementary aims. First, we describe monthly phenology for the full 50-species assemblage to show the strength of seasonal structure in the system [15]. Second, we use the full 50-species assemblage only to define the multiple-testing family for BH–FDR and to provide broader analytical context, whereas the focal13 subset serves as the main interpretive set for the heatmap, network summaries, and discussion.

Our hypotheses were intentionally conservative and framed as expectations about pattern structure rather than direct species interactions. We expected positive anomaly-based ecological associations to be more common within broad guilds whose members track similar habitat states, whereas negative anomaly-based ecological associations—if present—would be more likely among species that segregate in timing or habitat use [16,17]. We further expected de-seasonalization to reduce raw-count correlations driven mainly by shared phenology. These expectations concern patterns in the inferred association graph and do not, by themselves, identify mechanism [10,11].

### 1.5. Study Area and Timeframe

**Figure 1:**
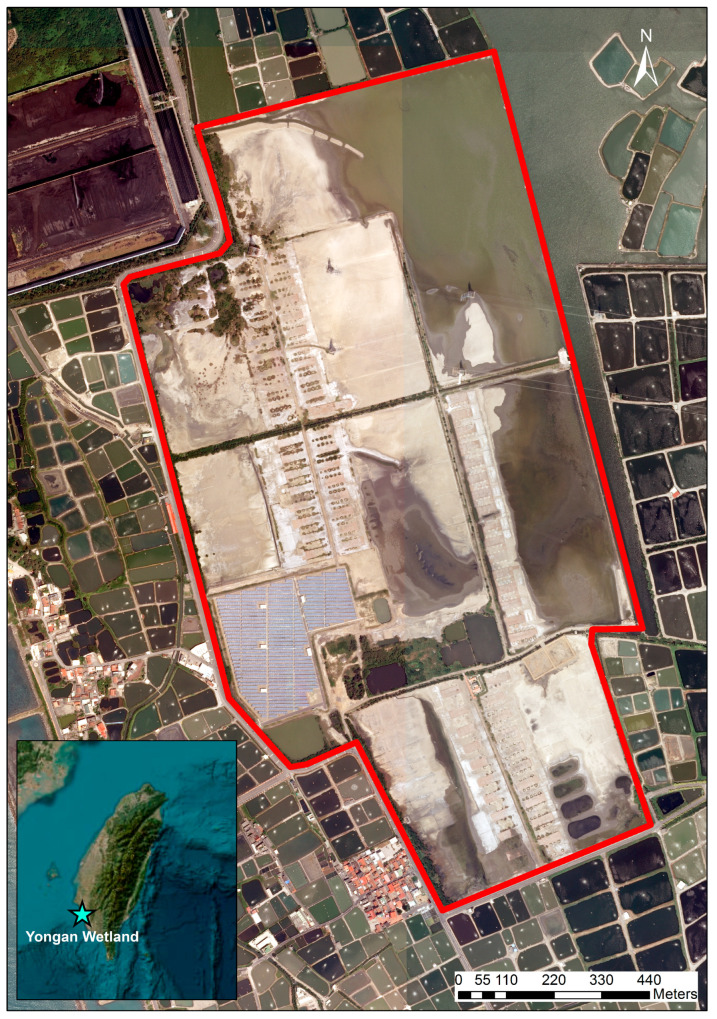
Location of the study area at Yongan Wetland (an abandoned saltpan wetland) in southwestern Taiwan. The red polygon delineates the standardized monthly waterbird survey area used for all time-series summaries and interspecific association analyses. The inset map shows the position of Yongan Wetland in Taiwan (star). North arrow and scale bar are shown.

Our analyses were conducted in Yongan Wetland, a ~130-ha coastal artificial wetland formed on abandoned saltpans in southwestern Taiwan, adjacent to the Hsinta Power Plant and embedded in an aquaculture-dominated landscape (Figure 1). The site comprises a mosaic of shallow impoundments and intermittently exposed flats that are predictably used by migratory waterbirds, providing a compact system in which repeated community snapshots can be interpreted against a stable landscape template.

We used monthly bird assemblage counts collected across two survey blocks totaling 36 standardized survey months (November 2014 and January–August 2015; October 2016–December 2018), with an intervening gap in standardized monthly monitoring between blocks. Because our inference targets seasonal migration dynamics and cross-species synchrony, we report seasonal-window patterns using the a priori migration windows (Section 2.2), while retaining the monthly record for anomaly calculation and anomaly-based association inference (Section 2.1, Section 2.2 and Section 2.3).

## 2. Material and Methods

### 2.1. Data and Preprocessing

Monthly bird assemblage data were derived from one standardized census date retained for each survey month. On that date, counts were conducted within 3.5 h after sunrise using fixed transects and fixed observation points across Yongan Wetland (Figure 1). Counts used flock-count–based scanning at vantage points, complemented by recording sporadic species encountered along the same transect-based survey framework, following widely used bird census principles for standardized field counting [18,19]. Yongan Wetland is a managed former saltpan wetland composed of shallow impoundments and exposed flats rather than an open tidal mudflat; however, as in any census-based monitoring framework, short-term redistribution of birds and temporal variation in detectability can still influence the number of individuals recorded on a given survey date. Bird identification, scientific nomenclature, and English common names followed AviList v2025, a unified global avian checklist, to ensure consistent and up-to-date taxonomy across years and analyses [20].

For the present analyses, we assembled a monthly species-by-time matrix comprising 36 survey months with standardized census data (Table S1). These surveys were collected in two blocks: November 2014 and January–August 2015 (*n* = 9; no survey in December 2014), and October 2016–December 2018 (*n* = 27), with no standardized monthly surveys between these blocks. Accordingly, the 36-month matrix used below should be interpreted as a standardized monthly monitoring record for interannual comparison and anomaly-based co-fluctuation analysis, rather than as a continuous day-by-day reconstruction of migration phenology.

**Figure 2:**
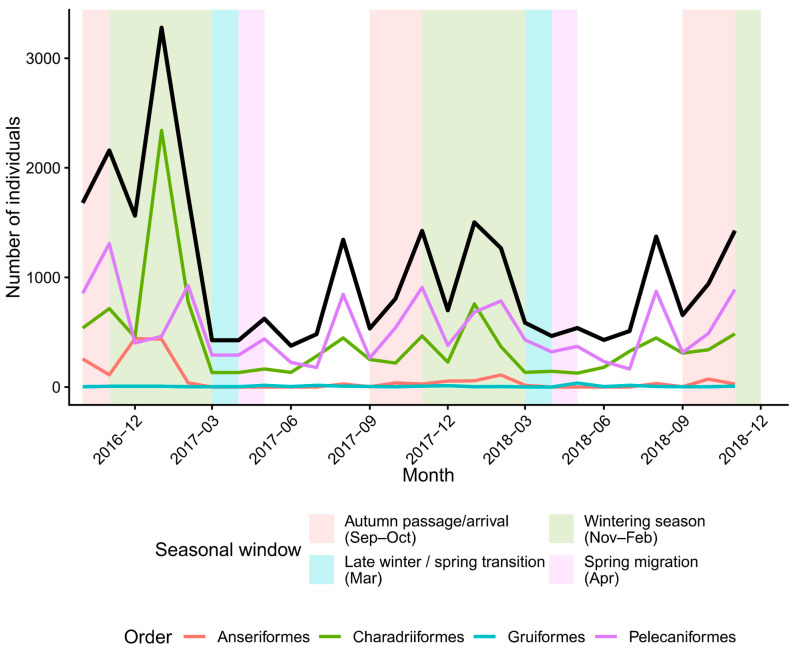
Monthly bird abundance dynamics during the later continuous survey block at Yongan Wetland (October 2016–December 2018). The black line shows the total number of individuals, and colored lines show order-level totals (Anseriformes, Charadriiformes, Gruiformes, and Pelecaniformes). Background shading indicates the predefined seasonal windows used for seasonal summaries (Table S4): autumn passage/arrival (Sep–Oct), wintering season (Nov–Feb), late winter/spring transition (Mar), and spring migration (Apr). This panel begins in October 2016 because it is intended to visualize the uninterrupted later block separately from the earlier block, which is summarized in Table S1 and represented in the species-level trajectories in Figure 3.

**Figure 3:**
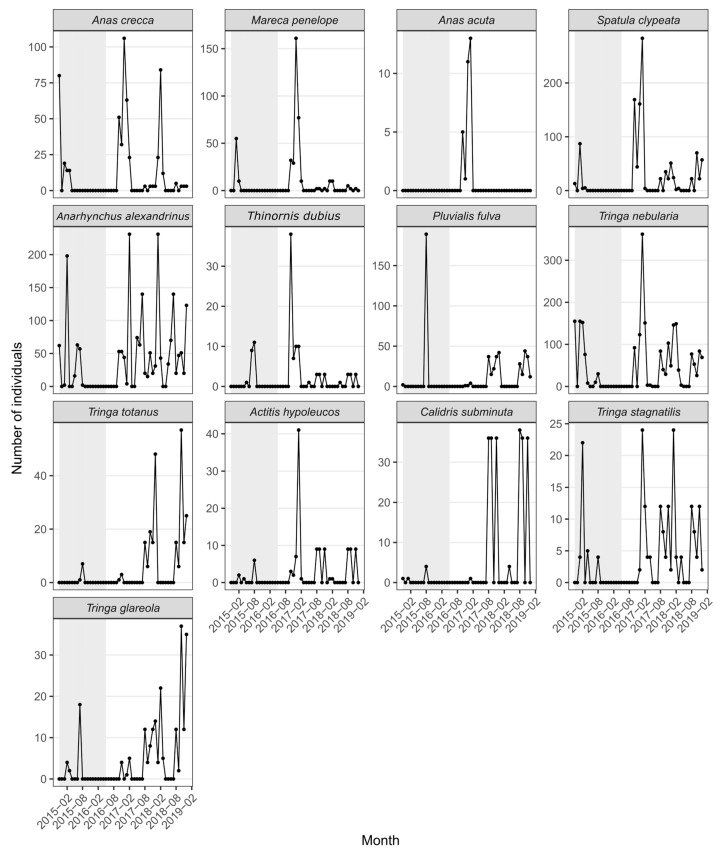
Representative species-level monthly count trajectories for the focal13 species, illustrating heterogeneous phenology across standardized monthly censuses. Points connected by lines show raw monthly counts on surveyed months; zero-valued points therefore indicate surveyed months in which the species was not recorded. The shaded interval marks the gap between the two standardized survey blocks, during which no standardized monthly surveys were conducted.

We considered two nested species sets. First, we compiled monthly counts for 50 regularly recorded waterbird species to summarize phenology and seasonal timing patterns (visualized in Figure 2, Figure 3 and Figure S1). For visual comparison of seasonal timing across many taxa, the phenology heatmap (Figure S1) used a per-species scaling of monthly abundance to the species-specific maximum (0–1), allowing peak-month structure to be compared without conflating differences in absolute abundance. Second, to test and interpret association patterns in detail, we defined a **focal13** subset (Table 1) comprising representative ducks (Anatidae) and shorebirds (Charadriidae/Scolopacidae) that were consistently recorded across the survey months and that span contrasting seasonal peaks. Table 1 lists the focal13 species, including common and scientific names, recorded months, and maximum monthly abundance.

### 2.2. Seasonal Windows Definition

We defined seasonal windows a priori based on local migratory phenology and assigned each month to one of the following categories: **Autumn passage/arrival (Sep–Oct)**, **Wintering (Nov–Feb)**, **Late winter/spring transition (Mar)**, and **Spring migration (Apr)** (Table S4). **May–Aug was treated as an off-season period outside the main migratory and wintering windows** and is **not discussed in the seasonal summaries** (e.g., phenology-focused summaries), consistent with the seasonal-window scheme.

### 2.3. Anomaly Definition/De-Seasonalization

To separate shared seasonal timing from departures around that timing, we analyzed two versions of each species’ monthly series: raw counts and anomalies [21]. Raw counts retain the full phenological signal and are used for descriptive summaries of timing and absolute abundance. Anomalies were calculated as the observed value minus the species-specific mean for that calendar month, so positive values indicate higher-than-typical abundance for that month and negative values indicate lower-than-typical abundance. For sensitivity analysis, we repeated the same procedure after applying log(count + 1) before calculating anomalies; this is referred to as the log-transformed anomaly analysis (Figure S4).

### 2.4. Association Inference

Pairwise ecological associations were quantified with Spearman’s rank correlation (ρ) [12]. We report correlations for both raw counts and anomaly series, but the main inferential focus is the anomaly series because it reduces shared phenological forcing. Off-season months (May–Aug) were retained in the monthly record to preserve temporal continuity, although they contribute little to migratory-season structure.

To control for multiple testing, we applied the Benjamini–Hochberg false discovery rate procedure across all 1225 pairwise tests in the 50-species assemblage [13,22]. We then display the focal13 subset as the main interpretive network. We use q ≤ 0.05 to define a conservative core of statistically supported associations; this threshold does not measure ecological strength or importance, and associations failing to pass it are not assumed to be absent.

### 2.5. Network Construction and Metrics

We translated the anomaly-based results into an undirected, weighted association network in which nodes represent species and edges represent focal13 pairs that remained significant after the global BH–FDR screen [23,24]. Edge weights were the corresponding Spearman correlations; positive edges were drawn as solid lines and negative edges as dashed lines [25,26]. The main network display is descriptive: it summarizes clustering, sign composition, and the relative concentration of associations within and between guilds [27,28].

Because our purpose is ecological interpretation rather than graph-theoretic optimization, we use network language conservatively and do not interpret graph position as evidence of direct interaction or causal influence [24,29]. The focal13 network is presented as a transparent summary of the conservative, thresholded association core.

### 2.6. Statistical Analysis

To assess robustness, we used three complementary diagnostics. First, we applied a moving-block bootstrap to the anomaly time series. The 36-month record was partitioned into contiguous 3-month blocks, and bootstrap replicates were generated by sampling those blocks with replacement and concatenating them to reconstruct pseudo-replicate series of the same length, thereby preserving short-range temporal dependence within each block (schematic in Figure S5A) [30,31,32]. For each of 1000 bootstrap replicates, we recalculated the full 50-species correlation matrix and reapplied BH–FDR across all 1225 tests; edge stability is reported as the proportion of replicates in which a focal13 edge again satisfied q ≤ 0.05 [31,32,33]. Unlike the circular-shift null comparison below, this bootstrap does not aim to destroy association structure; rather, it asks whether the observed edges are repeatedly recovered when the monthly record is reassembled from local temporal blocks.

Second, we used a circular-shift null comparison to ask whether the observed number of significant edges exceeded a conservative benchmark after cross-species alignment was disrupted (schematic in Figure S5B). For each species separately, its monthly anomaly series was shifted forward by a random cyclic lag, wrapping end values to the beginning, so that the within-species distribution and much of the temporal pattern were preserved while between-species temporal alignment was broken [34,35]. We generated 1000 such null realizations and recalculated the number of BH–FDR-significant edges each time. We treat this comparison as a heuristic benchmark rather than a perfect null model, because cyclic-shift procedures have known limitations [36].

Third, we repeated the anomaly analysis after log(count + 1) transformation to evaluate whether reducing the influence of extreme peaks changes the inferred associations. Together, these three checks address repeatability under block resampling, contrast against a conservative null benchmark, and sensitivity to transformation choice. None of them, by itself, establishes mechanism. All statistical analyses were conducted in R version 4.5.2 (R Foundation for Statistical Computing, Vienna, Austria). Network analyses/visualization were performed using igraph version 2.2.1, if applicable.

## 3. Results

### 3.1. Seasonal Dynamics and Phenology

Across the 36-month time series, the waterbird assemblage showed strong, repeatable seasonality, with abundance concentrated in the defined non-breeding period (Sep–Apr) and minimal occurrence during the off-season months (May–Aug). At the community level, total counts exhibited pronounced peaks during the wintering season (Nov–Feb) and consistent troughs outside the core non-breeding window (Figure 2). Order-level trajectories largely tracked the same seasonal envelope: Charadriiformes dominated the major winter peaks, with additional contributions from Pelecaniformes, whereas Anseriformes remained comparatively low and episodic, and Gruiformes contributed little throughout the series (Figure 2). This indicates that the assemblage-level signal is not driven by a single transient event, but reflects a recurring seasonal influx and redistribution across winters.

Species-level time series further revealed heterogeneous phenological profiles among focal taxa (Figure 3). Several focal species displayed sharp, pulse-like peaks restricted to one or a few months within the non-breeding season, consistent with migratory staging or short-duration stopover use. Other species showed broader winter presence spanning multiple consecutive months, consistent with overwintering residency in the system. A subset exhibited intermittent spikes (including occasional extreme peaks), highlighting that even within the same seasonal framework, taxa differ substantially in the timing, duration, and intensity of site use (Figure 3). Importantly, many long runs of zeros occurred during the off-season months (May–Aug) for multiple species, which reinforces that apparent co-occurrence in raw counts can be inflated by shared seasonality rather than indicating anomaly-based ecological association.

Extending beyond the focal set, the peak-month heatmap for 50 species summarized phenology in a standardized within-species scale (relative abundance 0–1), allowing direct comparison of timing while avoiding dominance by high-count species (Figure S1). Peak months were distributed across the annual cycle, but most species clustered in the non-breeding seasons, with distinct groups peaking in early winter, mid-winter, late winter/transition (Mar), or spring migration (Apr). Many taxa exhibited narrow peak-month signatures (single-month maxima), whereas others showed broader multi-month plateaus, indicating differing degrees of temporal specialization (Figure S1). Collectively, these results establish a clear phenological baseline for downstream association analyses: strong shared seasonality is pervasive at both order and species levels and must be accounted for when interpreting co-occurrence patterns.

### 3.2. Raw vs. Anomaly Association Contrast

We contrasted interspecific associations computed from **raw monthly counts** with those computed from **anomaly time series** in which within-year seasonality had been removed. In Figure S2, each point represents a species pair, plotted by raw Spearman’s ρ (x-axis) versus anomaly Spearman’s ρ (y-axis), with BH–FDR significance categories shown by symbol type.

**Figure 4:**
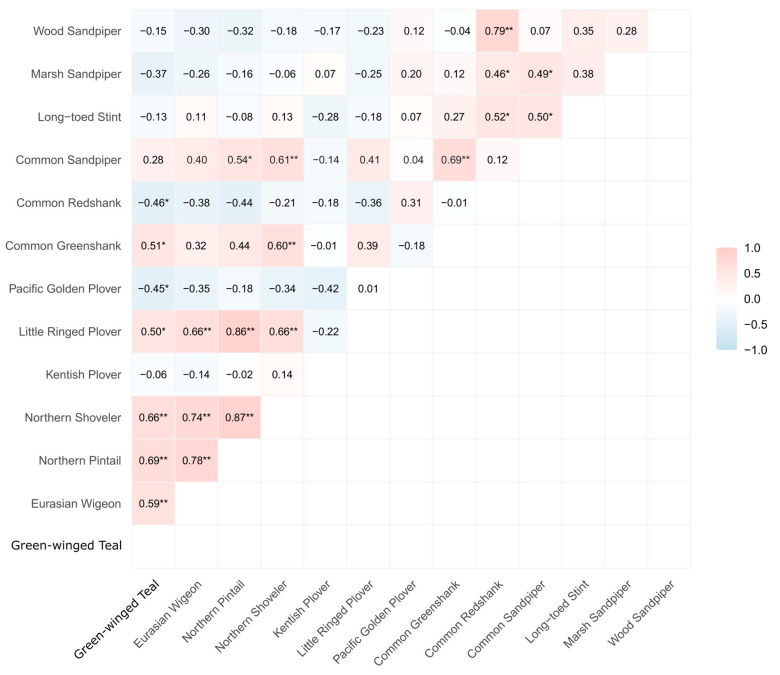
Interspecific associations among the focal13 species based on month-detrended raw-count anomalies (Spearman’s ρ; BH–FDR). BH–FDR was applied globally across all 1225 pairwise tests across the full assemblage of 50 species, and the focal13 subset is shown here for readability. Cell values are Spearman correlation coefficients computed on the month-detrended anomaly series, with colors indicating the sign and magnitude of ρ. Asterisks denote BH–FDR–adjusted significance (* q ≤ 0.05, ** q ≤ 0.01). Only one triangle of the symmetric matrix is displayed.

Overall, **most pairs clustered near the origin and were not significant in either analysis**, indicating weak or inconsistent covariation once pairwise comparisons were considered (Figure S2). A notable subset of associations was **significant only in raw counts** (pluses), but not after seasonality removal, consistent with correlations driven primarily by shared seasonal phenology rather than synchronous departures from seasonal baselines. The strongest raw-count significant pairs and their month-detrended (anomaly) counterparts are summarized in Table 2. Examples highlighted in Figure S2 include *Anas crecca–Tringa nebularia*, *Ardea cinerea–Himantopus himantopus*, *Phalacrocorax carbo–Pluvialis fulva*, and *Platalea minor–Tringa nebularia* (Figure S2). Across all 50 species (**BH–FDR across all 1225 tests**), raw monthly counts produced 140 significant associations (q ≤ 0.05), whereas month-detrended anomalies produced 164 (Figure S2; Tables S3 and S4). Within the focal13 subset (78 possible pairs), 23 pairs were significant in raw counts and 22 in anomalies; the overlap was incomplete (13 pairs significant in both, 10 raw-only, and 9 anomaly-only). Table 2 summarizes the 22 anomaly-supported focal13 edges (with their raw correlations shown for comparison), and Figure 4 visualizes these anomaly-based pairwise correlations as a heatmap; Table 2 also reports moving-block bootstrap edge stability.

**Figure 5:**
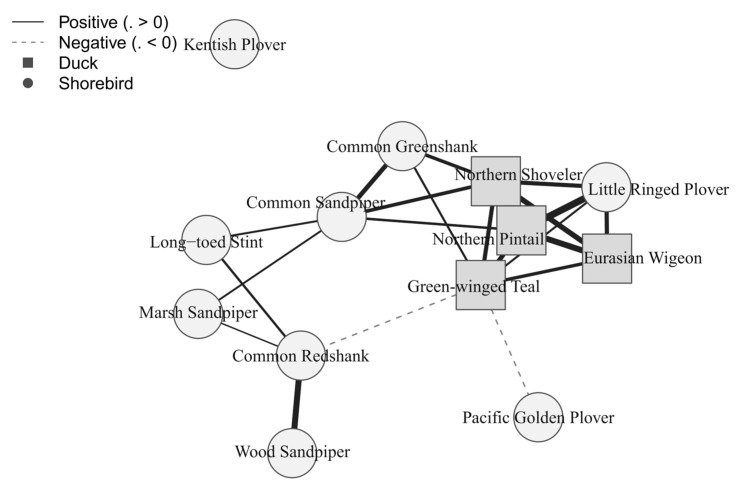
Association network for the focal13 subset derived from significant anomaly correlations (Spearman; BH–FDR q ≤ 0.05). Significance was determined using BH–FDR applied globally across the full assemblage of 50 species (1225 pairwise tests), and only the focal13 subset is visualized here. Nodes represent species; squares indicate dabbling ducks and circles indicate shorebirds. Solid edges indicate positive associations and dashed edges indicate negative associations; edge width is proportional to |ρ|. Only statistically supported links are displayed (q ≤ 0.05).

Conversely, several pairs became **significant only in the anomaly-based analysis** (circles), indicating covariation that is weak in raw series yet emerges when seasonal co-fluctuation is removed—i.e., **synchronous “above/below seasonal expectation” deviations**. Labeled examples include *Egretta garzetta–Platalea minor* and *Ardea cinerea–Sterna acuticauda* (Figure S2). Pairs significant in both analyses (triangles) were largely positive but generally **attenuated after seasonality removal** (points tending below the 1:1 line), suggesting that a component of their raw association was seasonal, while a residual non-seasonal coupling persisted (Figure S2). Collectively, Figure S2 supports using anomaly-based associations as the primary evidential layer for inferring interspecific co-dynamics beyond shared phenology.

### 3.3. Main Significant Associations Among the focal13 Species (Raw-Count Anomalies)

Using anomaly series for the focal13 species, we identified 22 significant associations after BH–FDR (Figure 4; Table S3). Of these, 20 were positive and 2 were negative. Significant edges occurred within ducks, within shorebirds, and between guilds, but the strongest and most cohesive structure involved the dabbling ducks and a smaller shorebird-centered cluster. Within the focal13 network, Little Ringed Plover formed the clearest bridge between the duck-dominated core and the broader shorebird assemblage, whereas the two negative edges both involved Green-winged Teal. Detailed coefficients are reported in Figure 4 and Table S3.

### 3.4. Network Structure of Significant Associations

Across the full 50-species assemblage, 164 of 1225 species pairs passed the BH–FDR threshold. In the main text, however, we focus on the focal13 subset because it is the main interpretive network discussed here. Within that subset, the anomaly-based network formed a single main component dominated by positive within-guild associations, especially among ducks, with fewer cross-guild links and only two retained negative edges (Figure 5).

### 3.5. Robustness and Sensitivity

#### 3.5.1. Edge Stability Under Moving-Block Bootstrap

Across 1000 moving-block bootstrap replicates, several focal13 edges were repeatedly recovered with high stability, especially within the dabbling-duck module and in a smaller subset of shorebird links (Figure S3A; Table 2). The most stable edges included Northern Pintail–Northern Shoveler and Little Ringed Plover–Northern Pintail, indicating that the core anomaly-based structure was not driven by a small subset of survey months.

#### 3.5.2. Circular-Shift Null Comparison

By contrast, the circular-shift null comparison yielded a median of 0 significant edges (range = 0–1) across 1000 null replicates, whereas the observed analysis retained 164 BH–FDR-significant associations across the full 50-species assemblage. Thus, when cross-species temporal alignment was disrupted while within-species temporal structure was largely preserved, the anomaly-based association signal collapsed (Figure S3B), supporting that the observed edge count is unlikely to arise from chance alignment alone.

#### 3.5.3. Sensitivity to Log Transformation

Most major guild-level features were retained under the log-transformed anomaly analysis, although some individual edges were transformation-sensitive (Figure S4). In particular, the positive duck module remained evident, whereas a subset of weaker cross-guild and negative edges changed under scale compression of large peaks. Taken together, these three diagnostics indicate that the main anomaly-based pattern is repeatable under block resampling, stronger than the circular-shift benchmark, and broadly robust to transformation choice.

## 4. Discussion

In this study, removing each species’ recurring seasonal baseline allowed us to quantify temporal co-fluctuation among migratory waterbirds at Yongan Wetland in southern Taiwan. The resulting ecological associations summarize how species covary after strong shared phenology has been reduced; they may reflect shared habitat tracking, synchronized responses to environmental conditions, or biotic processes, but they do not by themselves identify mechanism [8]. We therefore interpret the focal13 network as a conservative descriptive summary of community structure after shared phenology has been reduced, not as direct evidence of species interactions. Below, we first discuss the dominant guild structure (4.1), then consider negative associations cautiously (4.2), followed by robustness and limitations (4.3) and potential monitoring applications (4.4).

### 4.1. Ecological Interpretation: Guild Structure (Ducks vs. Shorebirds) and Shared Habitat/Foraging Regimes

A “guild” framing is useful when community patterns align more strongly with shared resource-use strategies than with taxonomy per se [16]. In our focal13 network, the four dabbling ducks (Green-winged Teal, Eurasian Wigeon, Northern Pintail, and Northern Shoveler) formed the most cohesive module, consistent with their broadly similar foraging mode and shallow-water reliance during wintering and passage. This type of within-guild cohesion is expected when species track the same habitat states (e.g., water depth and accessible benthic/epibenthic food) and respond synchronously to seasonal conditions.

Evidence from other systems supports this interpretation. For example, in a temporary guild of spring-staging dabbling ducks, Arzel and Elmberg (2004) [37] reported strong microhabitat-level convergence, with birds overusing shallow inshore areas relative to availability—exactly the kind of shared habitat tracking that can generate strong positive temporal associations at the monthly scale. In saltpan-derived wetlands, such shared tracking can be amplified because hydrological states and food availability can shift rapidly, and the set of “good” ponds may be simultaneously attractive to multiple dabbling duck species.

A second set of positive associations involved the shorebird assemblage, spanning plovers and sandpipers (including *Tringa* spp.). Shorebirds commonly concentrate in habitats where shallow water and exposed substrates make benthic prey accessible, and such conditions can promote simultaneous increases across multiple species even when they partition prey types or feeding microhabitats. For instance, trophic niche segregation within shorebird communities feeding on intertidal mudflats has been documented, but it occurs against the backdrop of shared dependence on the same broad habitat template [38].

In saltwork landscapes, this shared habitat template can extend beyond the natural intertidal zone. Classic work in Mediterranean saltworks showed that supratidal saltworks can function as important feeding habitat for waders, with behavioural evidence that birds may achieve higher feeding rates in saltworks ponds than on nearby mudflats, depending on conditions [39]. This supports a general mechanism for positive shorebird–shorebird associations in our system: when shallow ponds or exposed substrates become available, multiple wader species can respond together, even if they subsequently segregate at finer scales.

Finally, several robust cross-guild edges (ducks–shorebirds) are ecologically plausible in a saltpan mosaic where the same hydrological transition can simultaneously favour both guilds—for example, water drawdown that exposes soft substrates while maintaining shallow margins. Under such conditions, dabbling ducks can continue to exploit shallow water, while shorebirds expand onto newly exposed or marginal zones. We emphasize, however, that these cross-guild links are best interpreted as shared regime tracking at the landscape scale, not as direct facilitation, and they should motivate targeted follow-up work (e.g., coupling monthly counts with within-pond water-level states or spatial proximity metrics).

### 4.2. Negative Associations: Potential Niche Partitioning/Spatiotemporal Segregation (Bounded Interpretation)

In contrast to the dense core of positive within-guild links, only two focal13 edges remained negative after thresholding in the main anomaly analysis. We interpret this scarcity cautiously. It may partly reflect true asymmetry in how species track habitat states, but it may also reflect properties of the thresholded inference framework, including the greater difficulty of retaining negative correlations in short, noisy time series.

Ecologically, negative correlations in abundance anomalies can arise from several non-exclusive mechanisms. One possibility is niche differentiation in space or time—i.e., species using the same landscape but peaking under different water levels, substrate states, or seasonal windows [17,40]. Empirical work at stopover wetlands also shows that coexisting shorebirds can segregate along water-depth and foraging microhabitat gradients, producing predictable shifts in co-occurrence through time [41]. Another possibility is purely indirect: two species can appear negatively associated if they respond in opposite directions to the same (measured or unmeasured) environmental driver, or if detection and availability differ across habitats and seasons. Importantly, because our edges are inferred from observational co-occurrence time series rather than direct interaction data, negative associations should not be interpreted as evidence of competition or avoidance per se [8,42]. We therefore treat the negative links as hypotheses about potential partitioning or turnover that merit targeted follow-up, rather than as confirmed biotic interactions.

The most consistent negative pattern involved Pacific Golden Plover versus dabbling ducks (Green-winged Teal, Eurasian Wigeon, and Northern Shoveler) in the log-anomaly network. A parsimonious explanation is habitat-state switching within the saltpan mosaic: conditions that increase shallowly flooded foraging opportunities for dabbling ducks can simultaneously reduce the availability of exposed substrates favored by plovers, and vice versa. Under this view, the negative edges reflect alternating habitat templates—flooded versus exposed—rather than direct interference. Likewise, negative links between Northern Pintail (*Anas acuta*) and Tringa waders (*T. totanus*; *T. glareola*) may reflect seasonal turnover between wintering peaks of ducks and migratory peaks of shorebirds, consistent with temporal niche segregation as a stabilizing mechanism [40]. Such interpretations align with the broader expectation that coexistence in highly dynamic coastal wetlands can be supported by fine-scale partitioning across water depth, substrate, and phenological timing, but our current analysis cannot distinguish these pathways.

Overall, the limited number and transformation-sensitivity of negative edges reinforce a conservative conclusion: the dominant signal in the focal13 assemblage is shared positive tracking of habitat regimes. Under log transformation, the main duck-centered positive module remained, whereas some weaker negative or cross-guild edges changed, indicating that a small subset of edges is sensitive to the compression of large peaks. We therefore treat these transformation-sensitive edges as lower-confidence hypotheses for future validation using the point-level spatial proximity metrics available in our dataset (see Section 4.4).

### 4.3. Robustness and Limitations: Observational Time Series, Detection Effort, Remaining Confounders

Because our network inference is based on observational monthly time series, apparent associations can be shaped by temporal dependence, imperfect detection, and thresholding choices [31,32,43]. The robustness checks were therefore designed to address three limited questions: whether key edges are repeatedly recovered under block resampling, whether the observed number of edges exceeds a conservative null benchmark, and whether the main pattern is sensitive to log transformation. They were not intended to prove mechanism or to validate every retained edge individually.

The circular-shift comparison should also be interpreted cautiously. Although it provides a useful benchmark for disrupting cross-species alignment while retaining much of the within-species temporal pattern, cyclic-shift algorithms have known limitations and may distort aspects of temporal structure under some community-dynamics settings [34,35,36]. We therefore use the null result as heuristic support rather than as standalone proof.

More generally, correlation-based edges quantify statistical association, not direct interaction, and can be induced by shared habitat use, synchronized movement, unmeasured environmental drivers, or common detection conditions [8,44]. In addition, the monthly series used here are standardized census snapshots rather than continuous records, so they are better suited to describing broad seasonal structure and anomaly-based interspecific co-fluctuation than to resolving fine-scale migratory peaks or short-lived within-season turnover. Accordingly, the inferred edges should be interpreted as conservative, hypothesis-generating associations rather than exhaustive measures of instantaneous site use or mechanism.

### 4.4. Monitoring Implications and Future Directions

Although our anomaly-based associations should not be interpreted as direct biotic interactions, their **stability and recurrence** across resampled time blocks suggests they can still be useful as **monitoring signals** of community organization in a managed wetland [8]. In particular, the presence of a tightly connected positive core (e.g., within dabbling ducks) and seasonally aligned shorebird associations provides an interpretable summary of how the assemblage is structured in time, beyond changes in single-species abundance. From a monitoring perspective, a parsimonious set of **“sentinel edges”**—pairs that repeatedly appear significant under moving-block bootstrap—could be tracked through subsequent years as a compact indicator of whether major guild structure and seasonal coordination remain intact. Because detectability and effort can vary across surveys, interpreting edge turnover should remain conservative and ideally be complemented by models that explicitly acknowledge imperfect detection and observation processes [8,44].

Operationally, this framework can support routine biodiversity surveillance by highlighting whether (i) the duck-dominated positive core weakens or fragments, (ii) shorebird subnetworks shift in timing, or (iii) negative associations become more prevalent—patterns that may reflect changes in habitat availability, disturbance regimes, or other unmeasured drivers rather than true competitive exclusion. Importantly, we do not claim that any specific management action caused a given edge pattern; instead, we suggest that **association-network summaries** can serve as an additional layer of evidence for early warning and hypothesis generation in long-term monitoring programs.

Future work can directly strengthen inference by incorporating the **spatial proximity information** embedded in our point-location datasets. Rather than relying only on temporal co-fluctuation in abundance anomalies, one can quantify interspecific proximity (e.g., nearest-neighbor distances, cross-type Ripley’s K, or cross-type pair correlation functions) to test whether temporally associated pairs are also spatially co-located, and whether negative associations correspond to spatial segregation at relevant scales [45,46]. A complementary direction is to integrate environmental covariates and shared spatial random effects using joint species distribution models, allowing a clearer separation between shared habitat responses and residual association structure [47]. Together, these extensions would move the analysis from “time-series association” toward a more mechanistic synthesis that links co-occurrence dynamics to space use, habitat configuration, and seasonal habitat tracking.

## 5. Conclusions

Using anomaly-based time series, we inferred a stable, guild-structured association network among focal waterbird species, characterized by stronger positive connectivity within dabbling ducks and within shorebirds, and comparatively fewer cross-guild links. The main methodological contribution is that this anomaly framework, together with robustness checks (e.g., resampling- and null-based diagnostics), provides a practical route to separate shared seasonal forcing from persistent co-fluctuation patterns in long-term monitoring data. Future work should integrate individual-level spatial proximity metrics from point locations to test whether negative associations reflect fine-scale spatiotemporal segregation versus remaining environmental confounding, and to strengthen inference for monitoring and management applications.

## Figures and Tables

**Table 1:** Focal waterbird species used for interspecific association analyses, with their recorded months and maximum monthly abundance in standardized monthly censuses at Yongan Wetland, Taiwan.

Family	English Common Name	Scientific Name	Recorded Months (Month No.)	Max Monthly Abundance
Anatidae	Green-winged Teal	*Anas crecca*	1–3, 8, 10–12	106
Anatidae	Eurasian Wigeon	*Mareca penelope*	1, 2, 8–12	161
Anatidae	Northern Pintail	*Anas acuta*	1, 10–12	13
Anatidae	Northern Shoveler	*Spatula clypeata*	1–3, 8, 10–12	283
Charadriidae	Kentish Plover	*Anarhynchus alexandrinus*	1, 2, 5–12	231
Charadriidae	Little Ringed Plover	*Thinornis dubius*	1, 5, 7–12	38
Charadriidae	Pacific Golden Plover	*Pluvialis fulva*	8–12	189
Scolopacidae	Common Greenshank	*Tringa nebularia*	1–4, 8–12	362
Scolopacidae	Common Redshank	*Tringa totanus*	7–12	57
Scolopacidae	Common Sandpiper	*Actitis hypoleucos*	1, 2, 4, 8–12	41
Scolopacidae	Long-toed Stint	*Calidris subminuta*	1, 4, 8, 9, 11	38
Scolopacidae	Marsh Sandpiper	*Tringa stagnatilis*	1–4, 8–12	24
Scolopacidae	Wood Sandpiper	*Tringa glareola*	1–3, 7–12	37

Recorded months are calendar months (1–12) in which the species was recorded during the monitoring period. Max monthly abundance is the highest count recorded for that species in any single monthly census. Scientific names are italicized. Overall monthly totals and survey months are summarized in Table S1.

**Table 2:** Raw-count versus month-detrended (de-seasonalized) association estimates for the focal13 species subset.

Species 1	Species 2	Group	ρ (Raw Monthly)	q (BH-FDR, Raw)	ρ (Anomaly)	q (BH-FDR, Anomaly)	Stability (BootStrap)
Northern Shoveler	Northern Pintail	D–D	0.54	0.003	0.87	1.20 × 10^−9^	1.00
Little Ringed Plover	Northern Pintail	D–S	0.57	0.001	0.86	4.22 × 10^−9^	1.00
Wood Sandpiper	Common Redshank	S–S	0.67	<0.001	0.79	1.44 × 10^−6^	0.99
Northern Pintail	Eurasian Wigeon	D–D	0.59	0.001	0.78	2.02 × 10^−6^	0.95
Northern Shoveler	Eurasian Wigeon	D–D	0.59	0.001	0.74	2.28 × 10^−5^	0.93
Northern Pintail	Green-winged Teal	D–D	0.52	0.004	0.69	1.79 × 10^−4^	0.96
Common Sandpiper	Common Greenshank	S–S	0.53	0.004	0.69	1.96 × 10^−4^	0.96
Little Ringed Plover	Eurasian Wigeon	D–S	0.45	0.020	0.66	5.03 × 10^−4^	0.88
Northern Shoveler	Green-winged Teal	D–D	0.82	<0.001	0.66	5.32 × 10^−4^	0.94
Little Ringed Plover	Northern Shoveler	D–S	0.19	0.426	0.66	5.32 × 10^−4^	0.89
Common Sandpiper	Northern Shoveler	D–S	0.31	0.120	0.61	0.002	0.94
Common Greenshank	Northern Shoveler	D–S	0.66	<0.001	0.60	0.002	0.97
Eurasian Wigeon	Green-winged Teal	D–D	0.70	<0.001	0.59	0.003	0.79
Common Sandpiper	Northern Pintail	D–S	0.42	0.032	0.54	0.014	0.81
Long-toed Stint	Common Redshank	S–S	0.38	0.047	0.52	0.017	0.85
Common Greenshank	Green-winged Teal	D–S	0.78	<0.001	0.51	0.020	0.81
Little Ringed Plover	Green-winged Teal	D–S	0.10	0.692	0.50	0.023	0.68
Long-toed Stint	Common Sandpiper	S–S	0.62	<0.001	0.50	0.024	0.69
Marsh Sandpiper	Common Sandpiper	S–S	0.68	<0.001	0.49	0.025	0.71
Common Redshank	Green-winged Teal	D–S	0.02	0.919	−0.46	0.040	0.61
Marsh Sandpiper	Common Redshank	S–S	0.27	0.195	0.46	0.042	0.59
Pacific Golden Plover	Green-winged Teal	D–S	0.15	0.563	−0.45	0.043	0.59

For each listed pair, we report Spearman’s ρ and the corresponding BH–FDR-adjusted q-value across all 1225 species-pair tests for both raw monthly counts and month-detrended anomalies. Pairs shown are those significant in the anomaly analysis (q ≤ 0.05), which forms the basis of the focal13 network in Figure 5; raw-count statistics are included for comparison. Edge stability is the proportion of 1000 moving-block bootstrap replicates (3-month blocks) in which the same edge again met q ≤ 0.05 after re-evaluating the full set of 1225 tests. Group codes: D–D = within ducks, S–S = within shorebirds, D–S = cross-guild.

## Data Availability

The datasets supporting this study are provided as Supplementary Tables S1–S4 and Supplementary Data S1 and S2, and the data and code supporting the findings of this study are openly available in Zenodo at https://doi.org/10.5281/zenodo.18366473.

## Supplementary Materials

The following supporting information can be downloaded at: https://www.mdpi.com/article/10.3390/biology15070522/s1, Figure S1. Peak-month phenology heatmap for 50 species (within-species scaled relative abundance 0–1); Figure S2. Raw-count vs. anomaly association contrast for all species pairs (raw Spearman’s ρ vs. anomaly Spearman’s ρ; BH–FDR categories); Figure S3. Robustness checks: (A) edge stability under moving-block bootstrap; (B) null-model distribution of significant edge counts; Figure S4. Sensitivity analysis heatmap (log-transformed anomaly pipeline; focal13); Figure S5. Schematic illustration of the moving-block bootstrap and circular-shift null comparison used in this study; Table S1. Monthly bird counts for 50 species used in time-series analyses (36 monthly surveys); Table S2. Pairwise Spearman rank correlations among focal13 species based on raw monthly counts (ρ, p, BH–FDR q; N = 36 months); Table S3. Pairwise Spearman rank correlations among focal13 species based on de-seasonalized anomaly series (raw-count anomaly; ρ_resid, p, BH–FDR q; N = 36 months); Table S4. Definition of seasonal windows used in seasonal analyses (Sep–Oct, Nov–Feb, Mar, Apr; May–Aug excluded); Supplementary Data S1. Complete pairwise association results for the raw-anomaly pipeline (CSV); Supplementary Data S2. Complete pairwise association results for the log-transformed anomaly pipeline (CSV).
