# Supplementary material for: Disentangling Seasonality from Co-Occurrence: Anomaly-Driven Networks of Migratory Waterbirds
Source: Biology (Basel). 2026 Mar 25;15(7):522. doi: 10.3390/biology15070522 (PMC13071969; doi:10.3390/biology15070522)
Supplement: Supplementary file 1 [file biology-15-00522-s001.zip › Supplementary Information_final_author_approved_1.pdf]

Supplementary Information

Table S1. Monthly totals of waterbird abundance and species richness recorded in Yongan Wetland (Taiwan) during the long-term monitoring period (Nov 2014–Aug 2015; Oct 2016–Dec 2018).

Year-Month	Total Individuals	Total Species
2014-Nov	808	15
2015-Jan	1403	16
2015-Feb	1857	19
2015-Mar	1038	15
2015-Apr	482	14
2015-May	454	12
2015-Jun	402	9
2015-Jul	412	16
2015-Aug	664	18
2016-Oct	1680	20
2016-Nov	2158	26
2016-Dec	1564	25
2017-Jan	3278	26
2017-Feb	1742	19
2017-Mar	427	13
2017-Apr	427	13
2017-May	623	12
2017-Jun	376	8
2017-Jul	482	8
2017-Aug	1343	29
2017-Sep	534	25
2017-Oct	806	20
2017-Nov	1425	29
2017-Dec	700	20
2018-Jan	1502	19
2018-Feb	1267	20
2018-Mar	587	15
2018-Apr	466	14
2018-May	539	13
2018-Jun	430	9
2018-Jul	511	7

2018-Aug	1373	31
2018-Sep	657	25
2018-Oct	942	20
2018-Nov	1427	31
2018-Dec	902	21

Column descriptions
Year–Month: Month of survey, formatted as YYYY-Mmm (e.g., 2014-Nov).
Total Individuals: Total number of individual waterbirds recorded in the monthly survey (sum across all species).
Total Species: Total number of waterbird species recorded in the monthly survey (species richness).

Note: Only months with completed monthly surveys are listed; months not shown indicate that no standardized monthly census data were available for that month.

Abbreviations: None.

Table S2. Complete pairwise Spearman rank correlation results among the 13 focal waterbird species based on raw monthly abundance (N = 36 months). For

each species pair, Spearman's ρ, unadjusted p-value, and BH-FDR–adjusted q-value are reported.

Species 1	Species 2	Spearman's ρ (raw monthly counts)	p_value	q (BH-FDR)(raw)	Sig_q_0.05	PairID
Pacific Golden Plover	Common Redshank	0.85	0.000	0.000	Yes	Pacific Golden Plover__Common Redshank
Green-winged Teal	Northern Shoveler	0.82	0.000	0.000	Yes	Green-winged Teal__Northern Shoveler
Common Greenshank	Green-winged Teal	0.78	0.000	0.000	Yes	Green-winged Teal__Common Greenshank
Eurasian Wigeon	Common Sandpiper	0.74	0.000	0.000	Yes	Eurasian Wigeon__Common Sandpiper
Green-winged Teal	Eurasian Wigeon	0.70	0.000	0.000	Yes	Green-winged Teal__Eurasian Wigeon
Common Greenshank	Eurasian Wigeon	0.68	0.000	0.000	Yes	Eurasian Wigeon__Common Greenshank
Little Ringed Plover	Common Sandpiper	0.68	0.000	0.000	Yes	Little Ringed Plover__Common Sandpiper
Marsh Sandpiper	Common Sandpiper	0.68	0.000	0.000	Yes	Marsh Sandpiper__Common Sandpiper

Wood Sandpiper	Common Redshank	0.67	0.000	0.000	Yes	Common Redshank__Wood Sandpiper
Common Greenshank	Northern Shoveler	0.66	0.000	0.000	Yes	Common Greenshank__Northern Shoveler
Long-toed Stint	Common Sandpiper	0.62	0.000	0.000	Yes	Long-toed Stint__Common Sandpiper
Common Greenshank	Marsh Sandpiper	0.61	0.000	0.001	Yes	Marsh Sandpiper__Common Greenshank
Northern Pintail	Eurasian Wigeon	0.59	0.000	0.001	Yes	Northern Pintail__Eurasian Wigeon
Eurasian Wigeon	Northern Shoveler	0.59	0.000	0.001	Yes	Eurasian Wigeon__Northern Shoveler
Pacific Golden Plover	Long-toed Stint	0.59	0.000	0.001	Yes	Pacific Golden Plover__Long-toed Stint
Northern Pintail	Little Ringed Plover	0.57	0.000	0.001	Yes	Little Ringed Plover__Northern Pintail
Northern Pintail	Northern Shoveler	0.54	0.001	0.003	Yes	Northern Pintail__Northern Shoveler
Long-toed Stint	Marsh Sandpiper	0.53	0.001	0.004	Yes	Marsh Sandpiper__Long-toed Stint
Common Greenshank	Common Sandpiper	0.53	0.001	0.004	Yes	Common Greenshank__Common Sandpiper

Eurasian Wigeon	Marsh Sandpiper	0.52	0.001	0.004	Yes	Marsh Sandpiper__Eurasian Wigeon
Green-winged Teal	Northern Pintail	0.52	0.001	0.004	Yes	Green-winged Teal__Northern Pintail
Pacific Golden Plover	Wood Sandpiper	0.51	0.001	0.005	Yes	Pacific Golden Plover__Wood Sandpiper
Pacific Golden Plover	Common Sandpiper	0.49	0.002	0.008	Yes	Pacific Golden Plover__Common Sandpiper
Wood Sandpiper	Northern Shoveler	0.45	0.006	0.020	Yes	Northern Shoveler__Wood Sandpiper
Eurasian Wigeon	Little Ringed Plover	0.45	0.006	0.020	Yes	Little Ringed Plover__Eurasian Wigeon
Little Ringed Plover	Long-toed Stint	0.44	0.007	0.020	Yes	Little Ringed Plover__Long-toed Stint
Pacific Golden Plover	Northern Shoveler	0.44	0.008	0.022	Yes	Pacific Golden Plover__Northern Shoveler
Northern Pintail	Common Sandpiper	0.42	0.012	0.032	Yes	Northern Pintail__Common Sandpiper
Pacific Golden Plover	Little Ringed Plover	0.41	0.013	0.034	Yes	Little Ringed Plover__Pacific Golden Plover
Pacific Golden Plover	Marsh Sandpiper	0.41	0.013	0.035	Yes	Marsh Sandpiper__Pacific Golden Plover
Wood Sandpiper	Marsh Sandpiper	0.40	0.015	0.039	Yes	Marsh Sandpiper__Wood Sandpiper

Northern Shoveler	Common Redshank	0.40	0.016	0.039	Yes	Common Redshank__Northern Shoveler
Common Greenshank	Wood Sandpiper	0.39	0.020	0.047	Yes	Common Greenshank__Wood Sandpiper
Common Greenshank	Long-toed Stint	0.38	0.021	0.047	Yes	Long-toed Stint__Common Greenshank
Long-toed Stint	Common Redshank	0.38	0.021	0.047	Yes	Common Redshank__Long-toed Stint
Kentish Plover	Long-toed Stint	-0.37	0.026	0.057	No	Kentish Plover__Long-toed Stint
Little Ringed Plover	Common Redshank	0.35	0.034	0.072	No	Little Ringed Plover__Common Redshank
Green-winged Teal	Common Sandpiper	0.35	0.037	0.076	No	Green-winged Teal__Common Sandpiper
Wood Sandpiper	Green-winged Teal	0.35	0.039	0.078	No	Green-winged Teal__Wood Sandpiper
Common Sandpiper	Common Redshank	0.32	0.053	0.104	No	Common Redshank__Common Sandpiper
Northern Shoveler	Common Sandpiper	0.31	0.063	0.120	No	Northern Shoveler__Common Sandpiper

Eurasian Wigeon	Long-toed Stint	0.30	0.078	0.144	No	Eurasian Wigeon__Long-toed Stint
Pacific Golden Plover	Common Greenshank	0.29	0.091	0.165	No	Pacific Golden Plover__Common Greenshank
Northern Shoveler	Marsh Sandpiper	0.28	0.094	0.167	No	Marsh Sandpiper__Northern Shoveler
Wood Sandpiper	Common Sandpiper	0.27	0.115	0.195	No	Common Sandpiper__Wood Sandpiper
Marsh Sandpiper	Common Redshank	0.27	0.114	0.195	No	Marsh Sandpiper__Common Redshank
Green-winged Teal	Marsh Sandpiper	0.24	0.158	0.263	No	Green-winged Teal__Marsh Sandpiper
Common Greenshank	Northern Pintail	0.22	0.198	0.322	No	Northern Pintail__Common Greenshank
Wood Sandpiper	Eurasian Wigeon	0.20	0.253	0.403	No	Eurasian Wigeon__Wood Sandpiper
Northern Shoveler	Little Ringed Plover	0.19	0.273	0.426	No	Little Ringed Plover__Northern Shoveler
Kentish Plover	Marsh Sandpiper	-0.17	0.318	0.486	No	Marsh Sandpiper__Kentish Plover
Pacific Golden Plover	Kentish Plover	-0.15	0.385	0.563	No	Pacific Golden Plover__Kentish Plover
Pacific	Green-	0.15	0.390	0.563	No	Green-winged

Golden Plover	winged Teal					Teal__Pacific Golden Plover
Kentish Plover	Little Ringed Plover	-0.15	0.381	0.563	No	Little Ringed Plover__Kentish Plover
Wood Sandpiper	Northern Pintail	-0.14	0.406	0.565	No	Northern Pintail__Wood Sandpiper
Common Greenshank	Little Ringed Plover	0.14	0.413	0.565	No	Little Ringed Plover__Common Greenshank
Wood Sandpiper	Long-toed Stint	0.14	0.406	0.565	No	Long-toed Stint__Wood Sandpiper
Pacific Golden Plover	Eurasian Wigeon	0.13	0.463	0.623	No	Pacific Golden Plover__Eurasian Wigeon
Common Greenshank	Common Redshank	0.12	0.480	0.634	No	Common Redshank__Common Greenshank
Little Ringed Plover	Marsh Sandpiper	0.12	0.488	0.635	No	Marsh Sandpiper__Little Ringed Plover
Kentish Plover	Common Sandpiper	-0.12	0.500	0.640	No	Kentish Plover__Common Sandpiper
Kentish Plover	Green-winged Teal	0.10	0.545	0.686	No	Green-winged Teal__Kentish Plover
Green-winged Teal	Little Ringed Plover	0.10	0.559	0.692	No	Green-winged Teal__Little Ringed Plover
Kentish Plover	Wood Sandpiper	0.09	0.597	0.728	No	Kentish Plover__Wood Sandpiper

Northern Shoveler	Long-toed Stint	0.09	0.610	0.733	No	Long-toed Stint__Northern Shoveler
Kentish Plover	Eurasian Wigeon	0.08	0.630	0.745	No	Eurasian Wigeon__Kentish Plover
Pacific Golden Plover	Northern Pintail	0.07	0.679	0.779	No	Pacific Golden Plover__Northern Pintail
Northern Pintail	Long-toed Stint	-0.07	0.679	0.779	No	Northern Pintail__Long-toed Stint
Kentish Plover	Common Greenshank	-0.04	0.796	0.868	No	Kentish Plover__Common Greenshank
Kentish Plover	Northern Shoveler	0.04	0.800	0.868	No	Kentish Plover__Northern Shoveler
Wood Sandpiper	Little Ringed Plover	0.05	0.777	0.868	No	Little Ringed Plover__Wood Sandpiper
Northern Pintail	Marsh Sandpiper	-0.04	0.801	0.868	No	Marsh Sandpiper__Northern Pintail
Northern Pintail	Common Redshank	-0.04	0.825	0.881	No	Northern Pintail__Common Redshank
Kentish Plover	Common Redshank	-0.03	0.864	0.899	No	Common Redshank__Kentish Plover
Eurasian Wigeon	Common Redshank	0.03	0.860	0.899	No	Common Redshank__Eurasian Wigeon
Green-	Commo	0.02	0.895	0.919	No	Green-winged

winged Teal	n Redshank					Teal__Common Redshank
Green-winged Teal	Long-toed Stint	0.01	0.939	0.952	No	Green-winged Teal__Long-toed Stint
Kentish Plover	Northern Pintail	0.00	0.987	0.987	No	Northern Pintail__Kentish Plover

Abbreviations: ρ, Spearman's rank correlation coefficient; BH-FDR, Benjamini–Hochberg false discovery rate.

Table S3. Spearman's rank correlations (raw-count anomaly) among the focal 13 species with BH-FDR correction.

Species 1	Species 2	PairID	Spearman's ρ (raw-count anomaly)	p-value	q (BH-FDR)	Sig_q_0.05	Sign
Eurasian Wigeon	Green-winged Teal	Green-winged Teal__Eurasian Wigeon	0.59	1.30e-04	0.003	Yes	Positive
Northern Pintail	Green-winged Teal	Green-winged Teal__Northern Pintail	0.69	3.51e-06	1.79e-04	Yes	Positive
Northern Pintail	Eurasian Wigeon	Eurasian Wigeon__Northern Pintail	0.78	1.48e-08	2.02e-06	Yes	Positive

Northern Shoveler	Green-winged Teal	Green-winged Teal__Northern Shoveler	0.66	1.42e-05	5.32e-04	Yes	Positive
Northern Shoveler	Eurasian Wigeon	Eurasian Wigeon__Northern Shoveler	0.74	2.42e-07	2.28e-05	Yes	Positive
Northern Shoveler	Northern Pintail	Northern Pintail__Northern Shoveler	0.87	4.88e-12	1.20e-09	Yes	Positive
Kentish Plover	Green-winged Teal	Green-winged Teal__Kentish Plover	-0.06	0.749	1.000	No	Negative
Kentish Plover	Eurasian Wigeon	Eurasian Wigeon__Kentish Plover	-0.14	0.429	0.731	No	Negative
Kentish Plover	Northern Pintail	Northern Pintail__Kentish Plover	-0.02	0.902	1.000	No	Negative
Kentish Plover	Northern Shoveler	Northern Shoveler__Kentish Plover	0.14	0.421	0.723	No	Positive
Little Ringed Plover	Green-winged Teal	Green-winged Teal__Little Ringed Plover	0.50	0.002	0.023	Yes	Positive
Little Ringed Plover	Eurasian Wigeon	Eurasian Wigeon__Little Ringed Plover	0.66	1.27e-05	5.03e-04	Yes	Positive

Little Ringed Plover	Northern Pintail	Northern Pintail__Little Ringed Plover	0.86	2.07e-11	4.22e-09	Yes	Positive
Little Ringed Plover	Northern Shoveler	Northern Shoveler__Little Ringed Plover	0.66	1.43e-05	5.32e-04	Yes	Positive
Little Ringed Plover	Kentish Plover	Kentish Plover__Little Ringed Plover	-0.22	0.192	0.454	No	Negative
Pacific Golden Plover	Green-winged Teal	Green-winged Teal__Pacific Golden Plover	-0.45	0.005	0.043	Yes	Negative
Pacific Golden Plover	Eurasian Wigeon	Eurasian Wigeon__Pacific Golden Plover	-0.35	0.034	0.147	No	Negative
Pacific Golden Plover	Northern Pintail	Northern Pintail__Pacific Golden Plover	-0.18	0.292	0.594	No	Negative
Pacific Golden Plover	Northern Shoveler	Northern Shoveler__Pacific Golden Plover	-0.34	0.045	0.171	No	Negative
Pacific Golden Plover	Kentish Plover	Kentish Plover__Pacific Golden Plover	-0.42	0.012	0.072	No	Negative
Pacific Golden Plover	Little Ringed Plover	Little Ringed Plover__Pacific Golden Plover	0.01	0.975	1.000	No	Positive

Common Greenshank	Green-winged Teal	Green-winged Teal__Common Greenshank	0.51	0.001	0.020	Yes	Positive
Common Greenshank	Eurasian Wigeon	Eurasian Wigeon__Common Greenshank	0.32	0.057	0.204	No	Positive
Common Greenshank	Northern Pintail	Northern Pintail__Common Greenshank	0.44	0.007	0.051	No	Positive
Common Greenshank	Northern Shoveler	Northern Shoveler__Common Greenshank	0.60	9.46e-05	0.002	Yes	Positive
Common Greenshank	Kentish Plover	Kentish Plover__Common Greenshank	-0.01	0.942	1.000	No	Negative
Common Greenshank	Little Ringed Plover	Little Ringed Plover__Common Greenshank	0.39	0.020	0.103	No	Positive
Common Greenshank	Pacific Golden Plover	Pacific Golden Plover__Common Greenshank	-0.18	0.294	0.594	No	Negative
Common Redshank	Green-winged Teal	Green-winged Teal__Common Redshank	-0.46	0.005	0.040	Yes	Negative
Common Redshank	Eurasian Wigeon	Eurasian Wigeon__Common Redshank	-0.38	0.021	0.105	No	Negative

Common Redshank	Northern Pintail	Northern Pintail__Common Redshank	-0.44	0.007	0.054	No	Negative
Common Redshank	Northern Shoveler	Northern Shoveler__Common Redshank	-0.21	0.216	0.491	No	Negative
Common Redshank	Kentish Plover	Kentish Plover__Common Redshank	-0.18	0.286	0.590	No	Negative
Common Redshank	Little Ringed Plover	Little Ringed Plover__Common Redshank	-0.36	0.033	0.144	No	Negative
Common Redshank	Pacific Golden Plover	Pacific Golden Plover__Common Redshank	0.31	0.065	0.222	No	Positive
Common Redshank	Common Greenshank	Common Greenshank__Common Redshank	-0.01	0.945	1.000	No	Negative
Common Sandpiper	Green-winged Teal	Green-winged Teal__Common Sandpiper	0.28	0.097	0.295	No	Positive
Common Sandpiper	Eurasian Wigeon	Eurasian Wigeon__Common Sandpiper	0.40	0.015	0.085	No	Positive
Common Sandpiper	Northern Pintail	Northern Pintail__Common Sandpiper	0.54	7.55e-04	0.014	Yes	Positive

Common Sandpiper	Northern Shoveler	Northern Shoveler__Common Sandpiper	0.61	6.68e-05	0.002	Yes	Positive
Common Sandpiper	Kentish Plover	Kentish Plover__Common Sandpiper	-0.14	0.400	0.702	No	Negative
Common Sandpiper	Little Ringed Plover	Little Ringed Plover__Common Sandpiper	0.41	0.012	0.073	No	Positive
Common Sandpiper	Pacific Golden Plover	Pacific Golden Plover__Common Sandpiper	0.04	0.815	1.000	No	Positive
Common Sandpiper	Common Greenshank	Common Greenshank__Common Sandpiper	0.69	4.00e-06	1.96e-04	Yes	Positive
Common Sandpiper	Common Redshank	Common Redshank__Common Sandpiper	0.12	0.484	0.787	No	Positive
Long-toed Stint	Green-winged Teal	Green-winged Teal__Long-toed Stint	-0.13	0.447	0.746	No	Negative
Long-toed Stint	Eurasian Wigeon	Eurasian Wigeon__Long-toed Stint	0.11	0.517	0.819	No	Positive
Long-toed Stint	Northern Pintail	Northern Pintail__Long-toed Stint	-0.08	0.643	0.950	No	Negative

Long-toed Stint	Northern Shoveler	Northern Shoveler__Long-toed Stint	0.13	0.439	0.740	No	Positive
Long-toed Stint	Kentish Plover	Kentish Plover__Long-toed Stint	-0.28	0.102	0.303	No	Negative
Long-toed Stint	Little Ringed Plover	Little Ringed Plover__Long-toed Stint	-0.18	0.294	0.594	No	Negative
Long-toed Stint	Pacific Golden Plover	Pacific Golden Plover__Long-toed Stint	0.07	0.675	0.979	No	Positive
Long-toed Stint	Common Greenshank	Common Greenshank__Long-toed Stint	0.27	0.114	0.320	No	Positive
Long-toed Stint	Common Redshank	Common Redshank__Long-toed Stint	0.52	0.001	0.017	Yes	Positive
Long-toed Stint	Common Sandpiper	Common Sandpiper__Long-toed Stint	0.50	0.002	0.024	Yes	Positive
Marsh Sandpiper	Green-winged Teal	Green-winged Teal__Marsh Sandpiper	-0.37	0.024	0.118	No	Negative
Marsh Sandpiper	Eurasian Wigeon	Eurasian Wigeon__Marsh Sandpiper	-0.26	0.129	0.349	No	Negative

Marsh Sandpiper	Northern Pintail	Northern Pintail__Marsh Sandpiper	-0.16	0.348	0.647	No	Negative
Marsh Sandpiper	Northern Shoveler	Northern Shoveler__Marsh Sandpiper	-0.06	0.738	1.000	No	Negative
Marsh Sandpiper	Kentish Plover	Kentish Plover__Marsh Sandpiper	0.07	0.672	0.976	No	Positive
Marsh Sandpiper	Little Ringed Plover	Little Ringed Plover__Marsh Sandpiper	-0.25	0.140	0.369	No	Negative
Marsh Sandpiper	Pacific Golden Plover	Pacific Golden Plover__Marsh Sandpiper	0.20	0.231	0.520	No	Positive
Marsh Sandpiper	Common Greenshank	Common Greenshank__Marsh Sandpiper	0.12	0.496	0.799	No	Positive
Marsh Sandpiper	Common Redshank	Common Redshank__Marsh Sandpiper	0.46	0.005	0.042	Yes	Positive
Marsh Sandpiper	Common Sandpiper	Common Sandpiper__Marsh Sandpiper	0.49	0.002	0.025	Yes	Positive
Marsh Sandpiper	Long-toed Stint	Long-toed Stint__Marsh Sandpiper	0.38	0.023	0.114	No	Positive

Wood Sandpiper	Green-winged Teal	Green-winged Teal__Wood Sandpiper	-0.15	0.387	0.695	No	Negative
Wood Sandpiper	Eurasian Wigeon	Eurasian Wigeon__Wood Sandpiper	-0.30	0.074	0.239	No	Negative
Wood Sandpiper	Northern Pintail	Northern Pintail__Wood Sandpiper	-0.32	0.060	0.209	No	Negative
Wood Sandpiper	Northern Shoveler	Northern Shoveler__Wood Sandpiper	-0.18	0.293	0.594	No	Negative
Wood Sandpiper	Kentish Plover	Kentish Plover__Wood Sandpiper	-0.17	0.322	0.623	No	Negative
Wood Sandpiper	Little Ringed Plover	Little Ringed Plover__Wood Sandpiper	-0.23	0.177	0.427	No	Negative
Wood Sandpiper	Pacific Golden Plover	Pacific Golden Plover__Wood Sandpiper	0.12	0.503	0.805	No	Positive
Wood Sandpiper	Common Greenshank	Common Greenshank__Wood Sandpiper	-0.04	0.810	1.000	No	Negative
Wood Sandpiper	Common Redshank	Common Redshank__Wood Sandpiper	0.79	9.44e-09	1.44e-06	Yes	Positive

Wood Sandpiper	Common Sandpiper	Common Sandpiper__Wood Sandpiper	0.07	0.698	0.994	No	Positive
Wood Sandpiper	Long-toed Stint	Long-toed Stint__Wood Sandpiper	0.35	0.037	0.153	No	Positive
Wood Sandpiper	Marsh Sandpiper	Marsh Sandpiper__Wood Sandpiper	0.28	0.103	0.305	No	Positive

Table S3 — Column definitions

- Species 1: English common name of focal species 1 in the pair.

- Species 2: English common name of focal species 2 in the pair.

- PairID: unique identifier for the species pair, generated by concatenating the two English common names with a double underscore ("__"); ordering follows the table's export convention.

- Spearman's ρ (raw-count anomaly): Spearman's rank correlation coefficient (ρ) calculated using raw-count anomalies (i.e., de-seasonalized monthly abundance anomalies) across the study period (N = 36 months). Values range from −1 to +1; positive values indicate synchronous deviations, negative values indicate opposite deviations.

- p-value: two-sided p-value for Spearman's rank correlation test.

- q (BH-FDR): Benjamini–Hochberg false discovery rate (BH-FDR) adjusted p-value (q-value) across all pairwise tests.

- Sig_q_0.05: significance flag based on BH-FDR correction (Yes if $q < 0.05$; otherwise No).

- Sign: direction of association based on the sign of ρ (Positive/Negative).

Table S3 — Table note

Spearman’s rank correlations are reported for all pairwise combinations among the 13 focal species based on raw-count anomalies (N = 36 months). Both unadjusted p-values and BH-FDR–adjusted q-values are provided to account for multiple comparisons. English common names are used in this table.

Table S3 — Abbreviations

ρ, Spearman’s rank correlation coefficient; BH-FDR, Benjamini–Hochberg false discovery rate; p, unadjusted p-value; q, BH-FDR–adjusted p-value.

Table S4. Seasonal windows used in this study.

Seasonal windows were defined to match local migratory phenology. The remaining months (May–Aug) were treated as off-season.

Seasonal window	Months
Autumn passage/arrival	Sep–Oct
Wintering season	Nov–Feb
Late winter / spring transition	Mar
Spring migration	Apr
Off-season (non-migratory)	May–Aug

Table S4 — Column definitions

- Seasonal window: seasonal category defined a priori to match local migratory

phenology.

- Months: calendar months included in each seasonal window (ranges indicate inclusive month spans).

Table S4 — Table note

Seasonal windows were defined to match local migratory phenology. The remaining months (May–Aug) were treated as off-season (non-migratory).

Table S4 — Abbreviations

Sep, September; Oct, October; Nov, November; Dec, December; Jan, January; Feb, February; Mar, March; Apr, April; May, May; Jun, June; Jul, July; Aug, August.

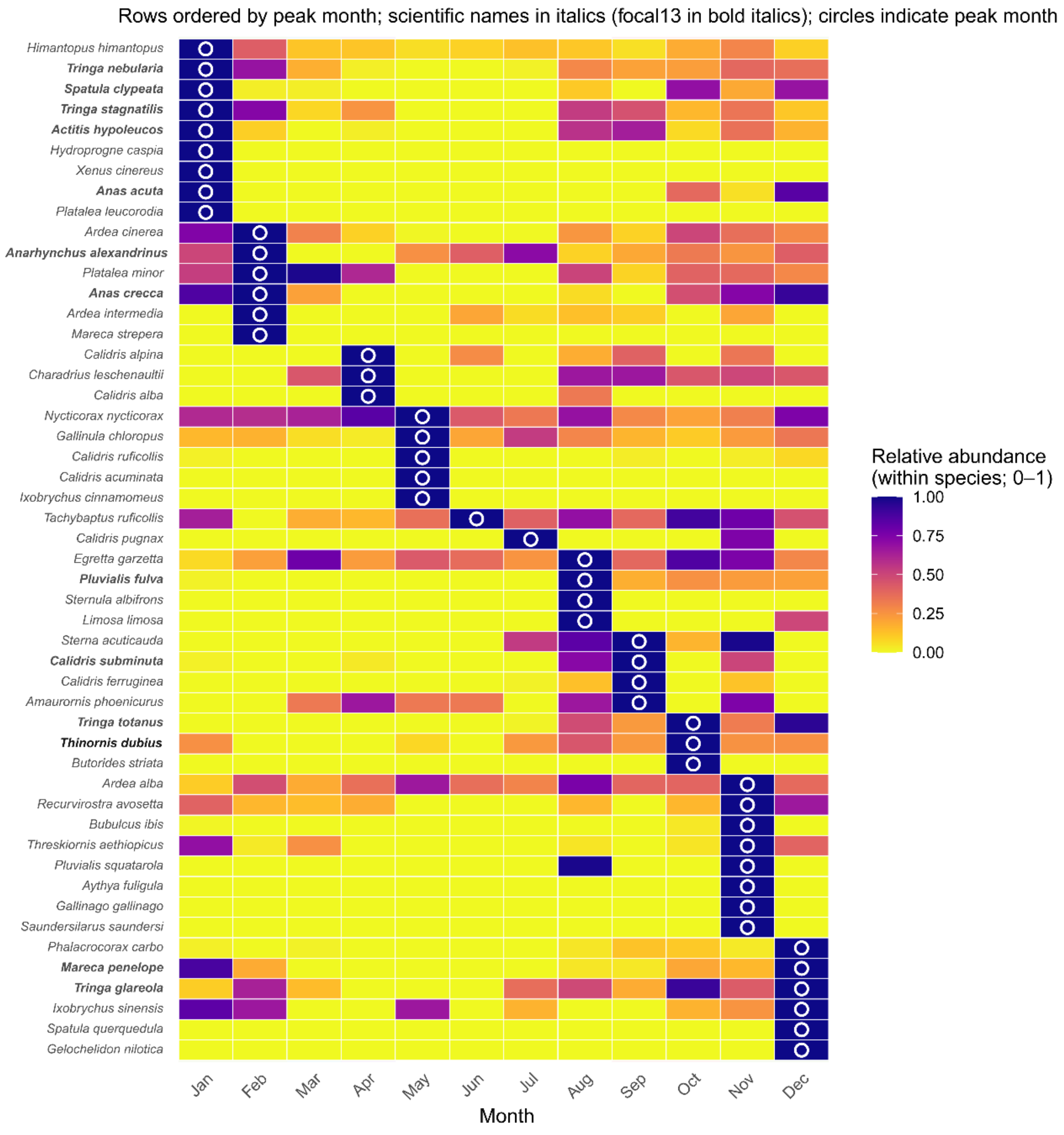

Figure S1. Seasonal peak-month heatmap for 50 species (phenology context for month-detrending).

Heatmap showing **within-species relative monthly abundance** (scaled 0–1 per species) across the calendar year (Jan–Dec), with rows ordered by each species' peak month. **Circles mark the peak month** for each species; scientific names are italicized, and **focal13 species are highlighted in bold italics**. This figure summarizes the strong month-of-year structure (phenology) present in the assemblage and provides context for the anomaly definition used throughout the association analyses (i.e., removing the month-of-year mean to focus on departures from seasonal baselines). For interpretation, seasonal windows follow the manuscript convention (e.g., Sep–Oct arrival, Nov–Feb wintering, Mar late-winter/spring transition, Apr spring migration), while the heatmap itself displays all months to visualize the complete seasonal cycle. Data are based on the same monthly survey series spanning **Nov 2014–Aug 2015 and Oct 2016–Dec 2018**.

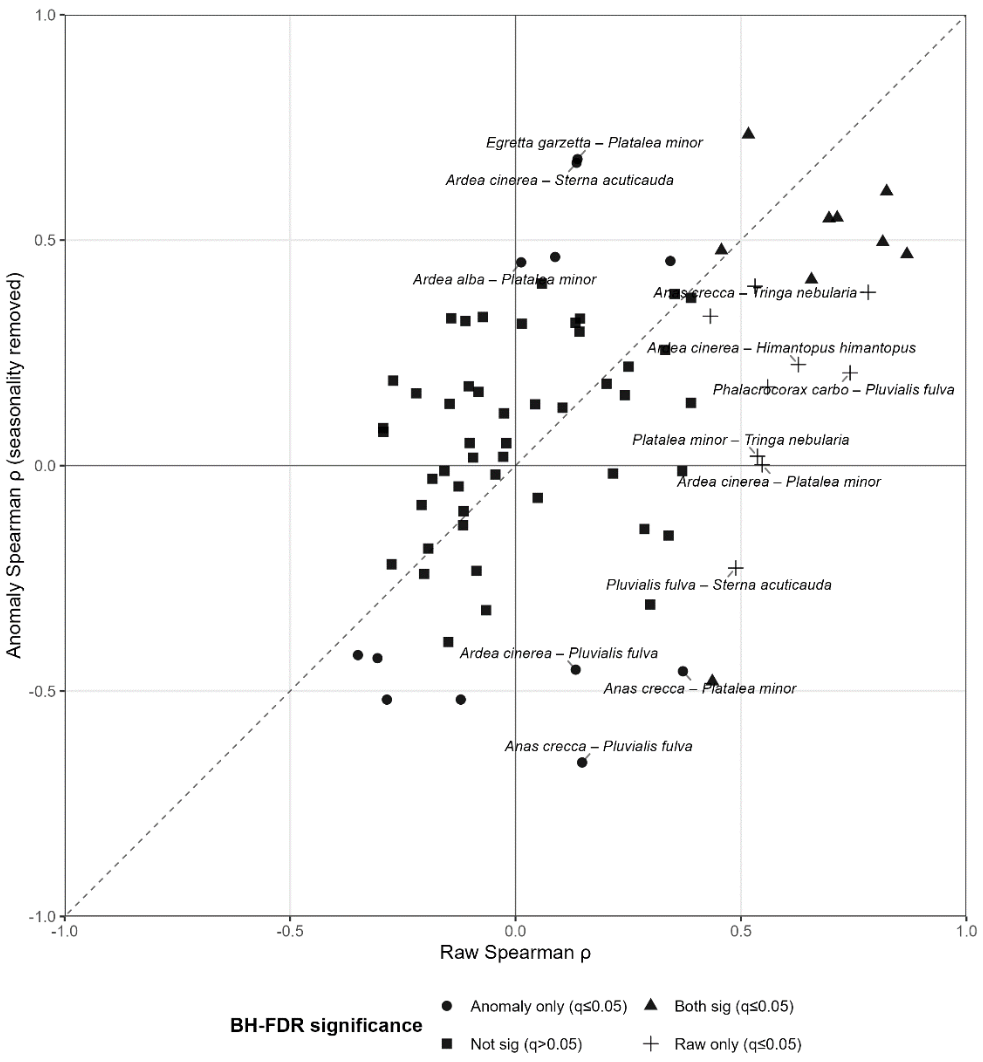

Figure S2. Comparison of association strength before and after de-seasonalization (50-species screen).

Scatterplot comparing Spearman rank correlations computed from (i) raw monthly counts (x-axis) versus (ii) month-detrended anomaly series (y-axis; raw-count anomalies with the month-of-year mean removed). Each point represents one of the **1,225 species pairs (50 choose 2)** from the full screening set. Points are categorized by BH–FDR significance ($q \leq 0.05$) evaluated **separately for the raw-count and anomaly-based tests**, highlighting pairs that are significant only in raw data (likely seasonality-driven), only after de-seasonalization (revealed co-variation in departures from seasonal baselines), significant in both, or not significant. The dashed line is the 1:1 reference (raw ρ = anomaly ρ). Monthly series were assembled from the available survey months spanning **Nov 2014–Aug 2015 and Oct 2016–Dec 2018**.

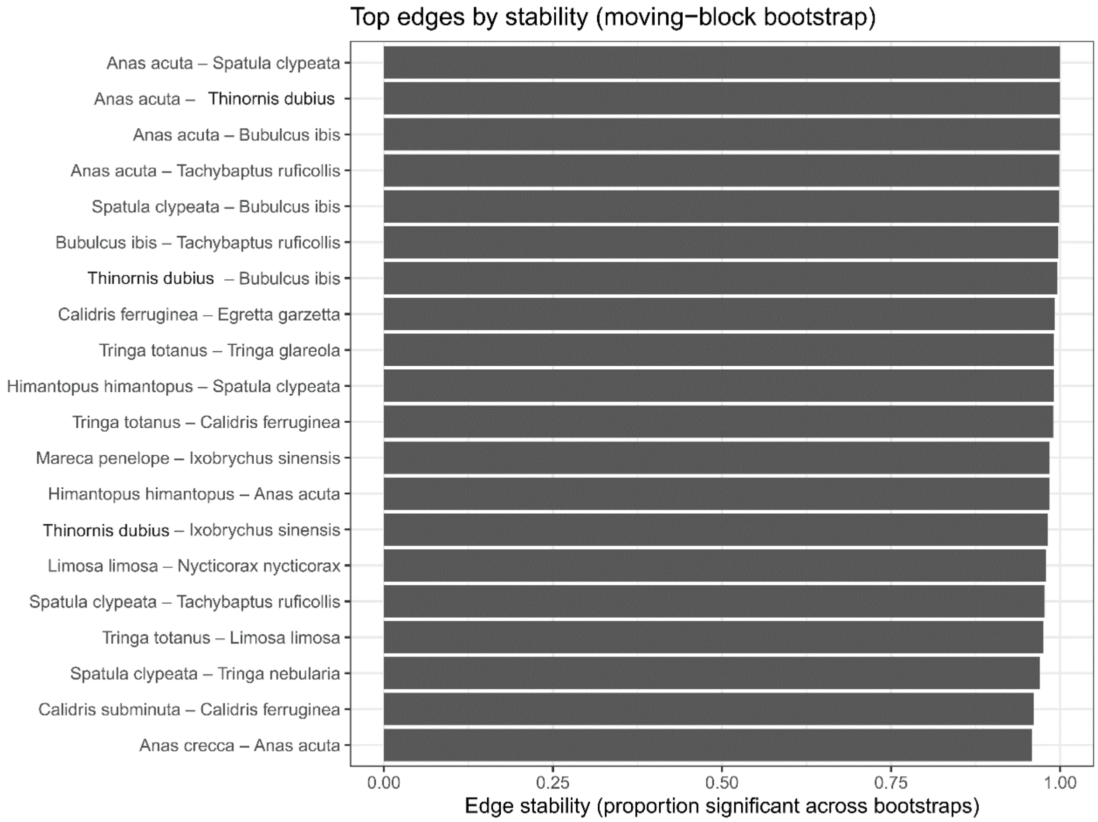

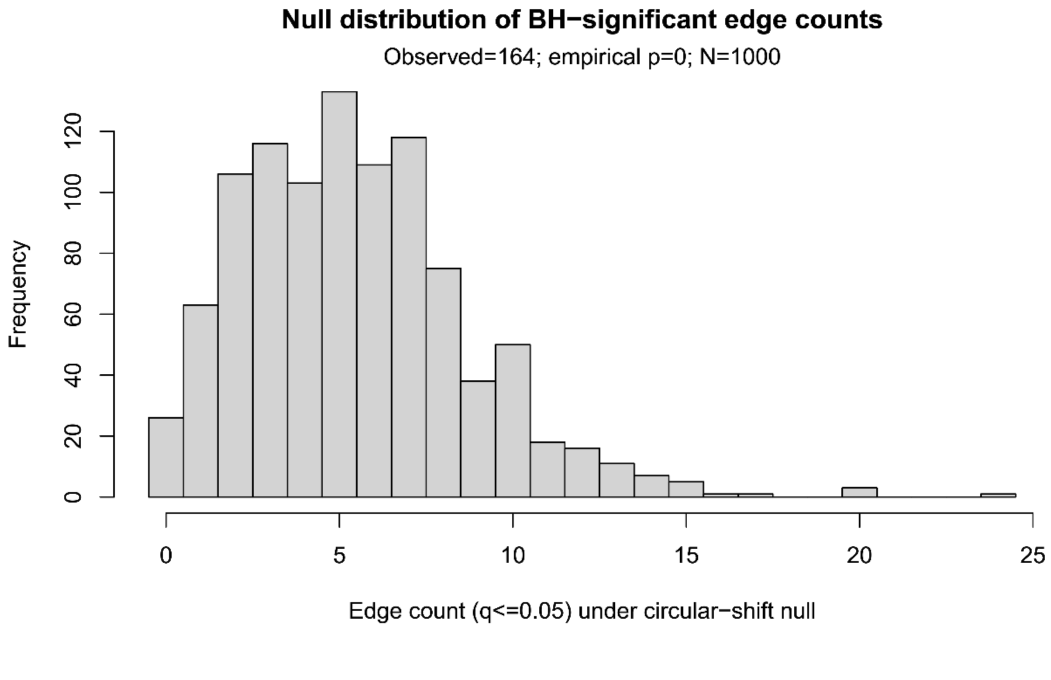

Figure S3. Robustness checks for the global BH–FDR association screen (moving-block bootstrap and circular-shift null). (A) Edge stability (moving-block bootstrap). For each bootstrap replicate (1,000 replicates; block length = 3 months to preserve short-term autocorrelation), we re-estimated Spearman correlations on the

anomaly series and re-applied BH–FDR across the full 1,225 tests. “Edge stability” is the proportion of bootstrap replicates in which a given observed significant association remained significant (q ≤ 0.05; same sign), and the plot shows the most stable edges. **(B)** Null distribution of the number of significant edges (circular-shift null). To test whether the observed network density could arise from shared seasonality/autocorrelation alone, we generated 1,000 null datasets by circularly shifting each species’ monthly series (shifted independently by species), then repeated the full anomaly-based Spearman screening with BH–FDR across 1,225 tests. The histogram shows the null distribution of the number of significant edges (q ≤ 0.05); the observed count (164 significant edges) is indicated for comparison. Monthly series were based on the same survey-month coverage (Nov 2014–Aug 2015 and Oct 2016–Dec 2018).

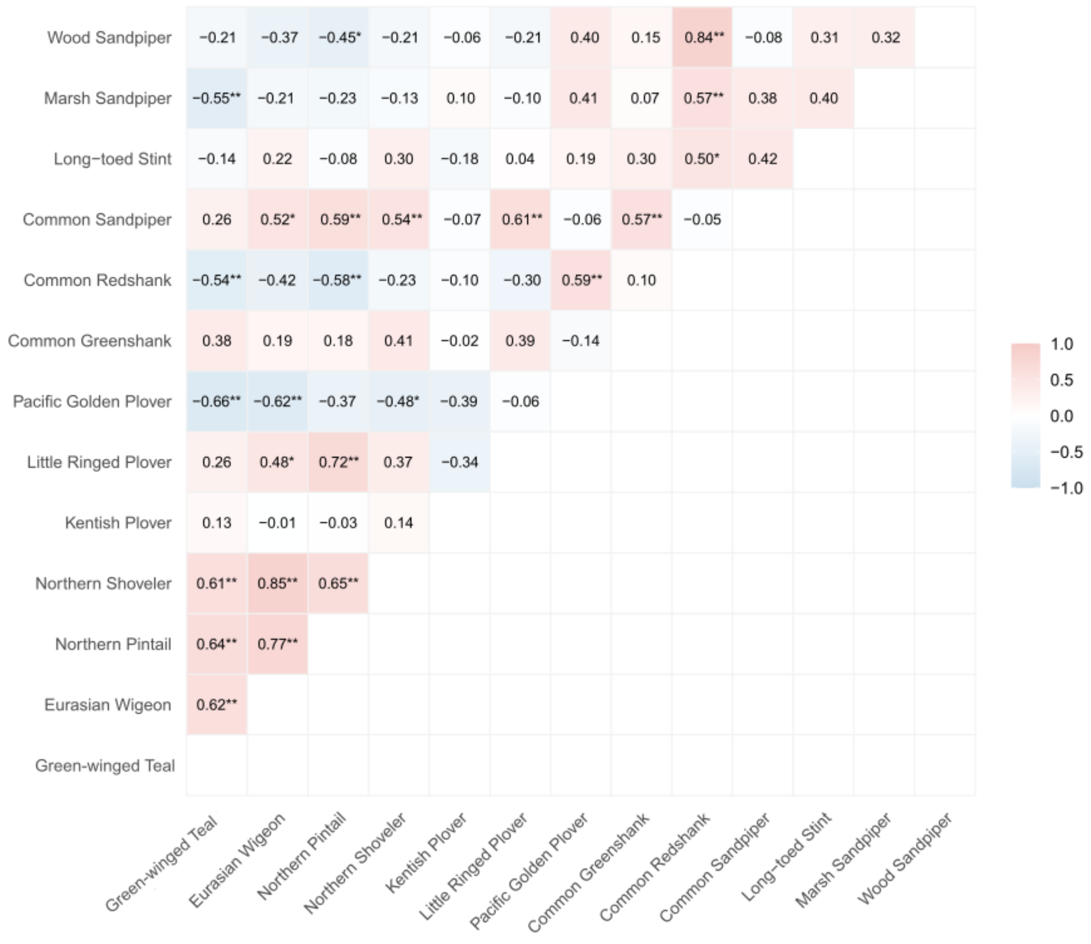

Figure S4. Sensitivity analysis: interspecific associations using log-transformed anomaly series (focal13 shown; global BH–FDR). Heatmap of pairwise Spearman correlations among the focal13 species, computed from log-transformed monthly

counts [log(count + 1)] followed by month-of-year detrending (log-transformed anomaly = log(count + 1) − mean log(count + 1) for that month-of-year). Cell values report Spearman's ρ; colors indicate direction and magnitude (positive vs negative associations). Statistical significance (asterisks) reflects BH–FDR correction applied over the full **50-species screen (1,225 pairwise tests)** using the same log-transformed anomaly pipeline, with the focal13 subset displayed here for readability. This sensitivity check evaluates whether the main anomaly-based conclusions are robust to a variance-stabilizing transformation that down-weights extreme abundance peaks. Monthly series used the full available survey months **(Nov 2014–Aug 2015 and Oct 2016–Dec 2018)**.

Figure S5. Schematic illustration of the two robustness resampling procedures

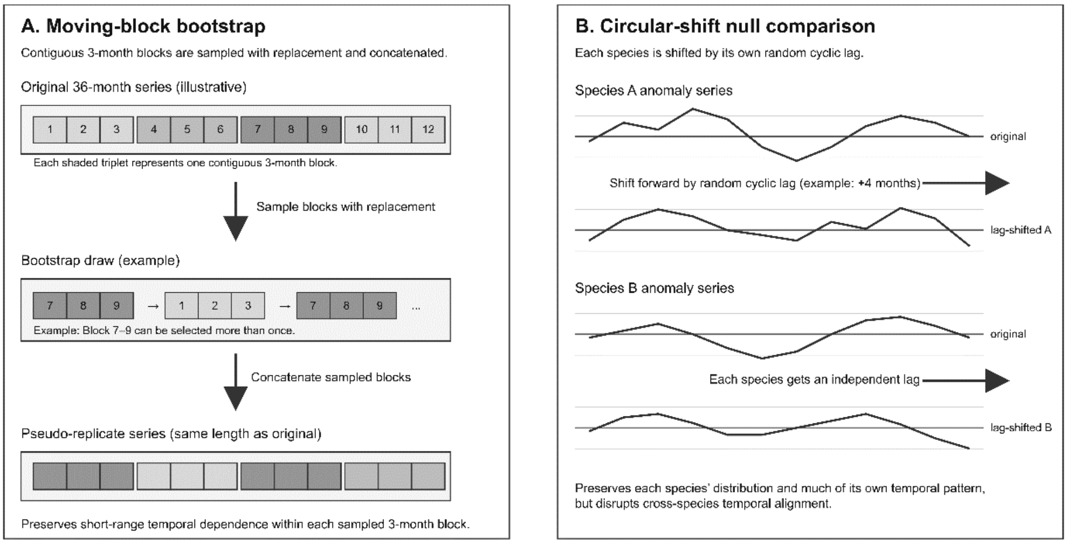

Figure S5. Schematic illustration of the two resampling-based robustness procedures used in this study. (A) Moving-block bootstrap: the 36-month record is partitioned into contiguous 3-month blocks, blocks are sampled with replacement, and the sampled blocks are concatenated to reconstruct a pseudo-replicate series of the same length. This preserves short-range temporal dependence within each sampled block while testing whether focal associations are repeatedly recovered when the monthly record is reassembled from local temporal segments. (B) Circular-shift null

comparison: each species' anomaly series is shifted forward by an independently selected random cyclic lag, wrapping end values to the beginning. This preserves the within-species distribution and much of the temporal pattern, while disrupting cross-species temporal alignment. The figure is conceptual and is included to clarify what is resampled or shifted in the two robustness procedures.
