# Supplementary figures and images for: Disentangling Seasonality from Co-Occurrence: Anomaly-Driven Networks of Migratory Waterbirds
Source: Biology (Basel). 2026 Mar 25;15(7):522. doi: 10.3390/biology15070522 (PMC13071969; doi:10.3390/biology15070522)

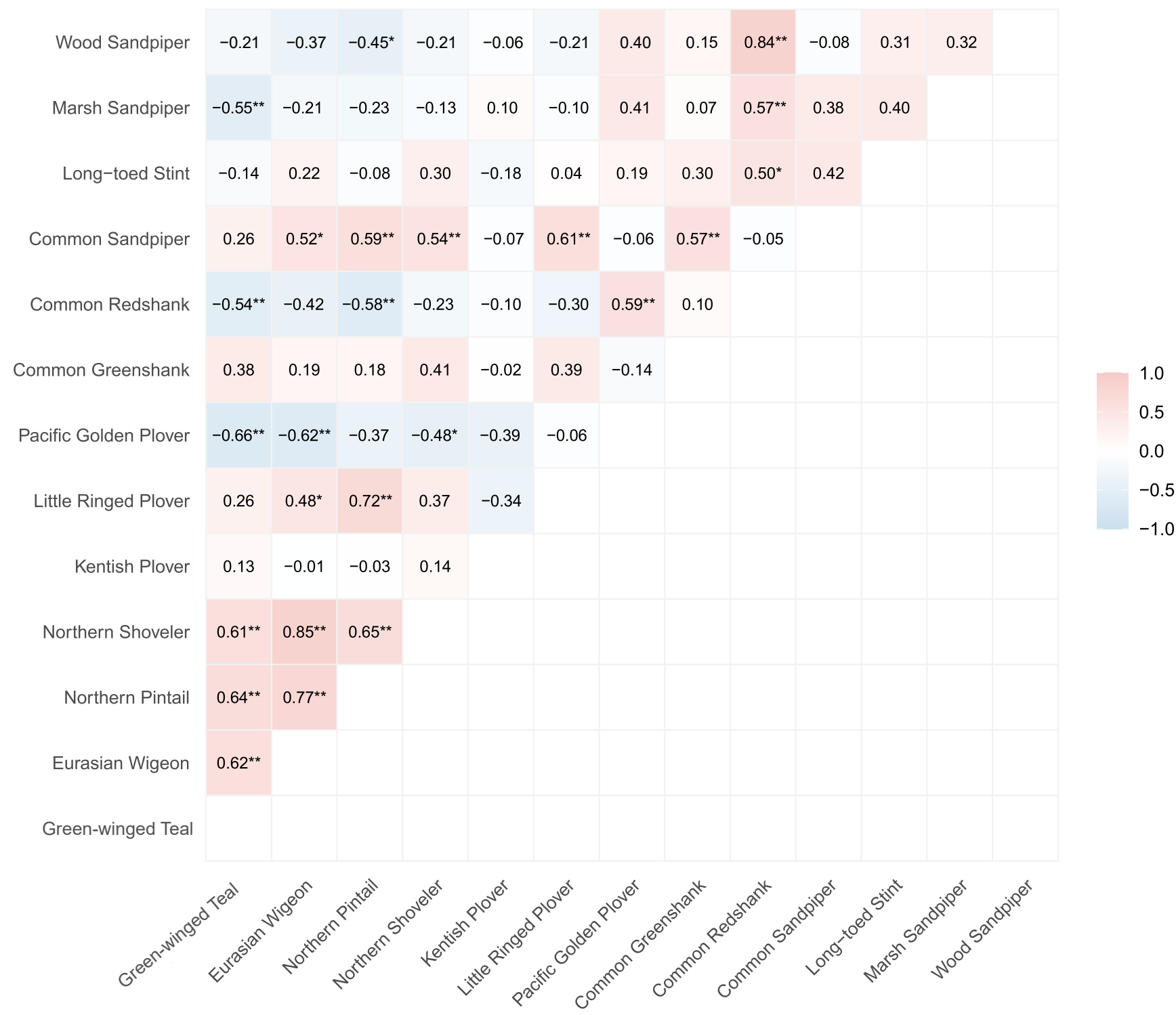

Supplement: Supplementary file 1 [file biology-15-00522-s001.zip › Figure S4_revised.pdf]

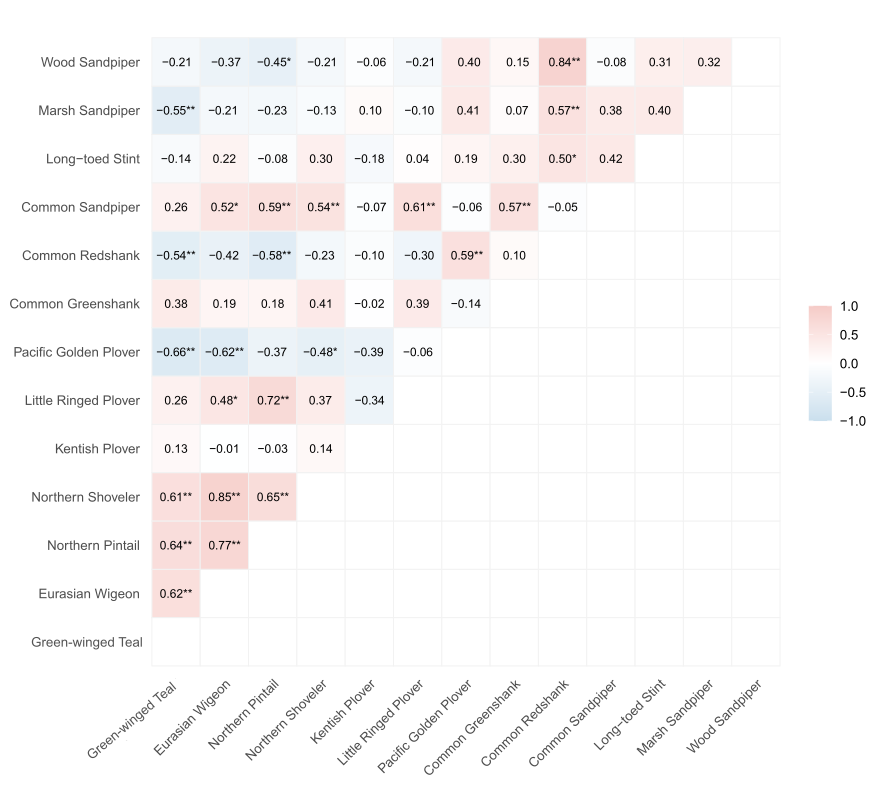

Supplement: Supplementary file 1 [file biology-15-00522-s001.zip › Figure S4_revised.png]
